# High-Mobility and High-Optical Quality Atomically Thin WS_**2**_

**DOI:** 10.1038/s41598-017-14928-2

**Published:** 2017-11-02

**Authors:** Francesco Reale, Pawel Palczynski, Iddo Amit, Gareth F. Jones, Jake D. Mehew, Agnes Bacon, Na Ni, Peter C. Sherrell, Stefano Agnoli, Monica F. Craciun, Saverio Russo, Cecilia Mattevi

**Affiliations:** 1 Department of Materials, Imperial College, London, SW7 2AZ UK; 2 Centre for Graphene Science, Department of Physics, University of Exeter, Stocker Road, Exeter, EX4 4QL UK; 3 Department of Chemical Sciences, University of Padua, Via F. Marzolo 1, 35131 Padua, Italy; 4 Centre for Graphene Science, Department of Engineering, University of Exeter, North Park Road, Exeter, EX4 4QF UK

## Abstract

The rise of atomically thin materials has the potential to enable a paradigm shift in modern technologies by introducing multi-functional materials in the semiconductor industry. To date the growth of high quality atomically thin semiconductors (e.g. WS_2_) is one of the most pressing challenges to unleash the potential of these materials and the growth of mono- or bi-layers with high crystal quality is yet to see its full realization. Here, we show that the novel use of molecular precursors in the controlled synthesis of mono- and bi-layer WS_2_ leads to superior material quality compared to the widely used direct sulfidization of WO_3_-based precursors. Record high room temperature charge carrier mobility up to 52 cm^2^/Vs and ultra-sharp photoluminescence linewidth of just 36 meV over submillimeter areas demonstrate that the quality of this material supersedes also that of naturally occurring materials. By exploiting surface diffusion kinetics of W and S species adsorbed onto a substrate, a deterministic layer thickness control has also been achieved promoting the design of scalable synthesis routes.

## Introduction

Atomically thin layers of metal group VI disulfides and diselenides (MoS_2_, WS_2,_ WSe_2_, MoSe_2_,) are being extensively investigated as they present unconventional optoelectronic properties compared to commonly used low-dimensional semiconductors^1,2^. In the bulk form they are layered compounds formed of covalently bonded chalcogen and metal atoms forming tri-atomic layers, which are held together by van der Waals forces^3^. An individual tri-atom thick layer presents a direct band gap in the visible-near IR range^4^ on the contrary to the bulk, which manifests an indirect electronic band gap. Monolayer sulfides and selenides show strong light absorption from the visible to the near IR range^1,5,6^, valley polarization^7,8^, second-harmonic generation^9^, tightly bound excitons^10^ and strong spin-orbit interaction^11,12^. These properties arise from their intrinsic two-dimensional nature inherently free from dangling bonds and their particular d-orbitals configuration^3,13^. Further, given their atomically thin nature they are mechanically flexible and they can sustain tensile strain of 20%^14,15^.

One of the most promising transition metal dichalcogenides (TMDCs) is WS_2_ owing to light emission in the monolayer form at ~2 eV and the low level of toxicity of growth processes. Any envisioned application relies on materials with high crystal and optical quality extended over wafer-size areas. Chemical vapour deposition (CVD) is a scalable method for materials synthesis and it is being widely employed for TMDCs^16,17^. The synthesis of tungsten-based materials has revealed to be challenging and generally leading to isolated flakes of lateral size between 5–40 μm^4,18–23^. Monolayer WS_2_ films extended over centimeter-sized areas has been demonstrated^24,25^, however with compromised crystal quality as indicated by the low carrier mobilities. The growth is typically performed by co-evaporating sulfur powder and a W-precursor in a horizontal tubular furnace in presence of a carrier gas. Until now, the synthesis of WS_2_ has been achieved predominantly using WO_3_ and S powders^4,18–23^ at temperatures greater than 900 °C. This involves a topotactic-like transformation^26^ which normally yields to sparsely distributed WS_2_ domains onto an amorphous^4,18,20,22,23^ or crystalline substrate^19,21,27^.

Here we demonstrate the synthesis of high quality monolayer WS_2_ using carbon-free molecular precursors. The high crystal quality is manifested by the record high charge carrier mobilities of mono- and bi-layer WS_2_ and ultra-sharp PL linewidth at room temperature, which are superior to those of naturally occurring materials. The growth is enabled by molecular precursors, which lead to a complete sulfidization of W and formation of WS_2_ with lower number of defects compared to the traditionally used direct sulfidization of WO_3_.

## Results and Discussion

The synthesis of WS_2_ was performed starting from commercial powders of either H_2_WO_4_ (hydrated tungsten oxide) or WO_3_, sulfur and where indicated, we have introduced NaCl. W and S precursors were placed in two separate crucibles well-spaced in a quartz tubular furnace (Supporting Information, Figure S1a) and heated up independently using different controllers as reported in Figure S1b. The heating profile of S powders has been optimized to ensure maximum supply when the W-precursors start evaporating. WS_2_ was grown on Si/SiO_2_ (285 nm) substrates loaded in the downstream zone of the tubular furnace. Altering the chemistry of decomposition of tungsten oxide species, we could achieve synthesis of monolayer WS_2_ over larger area coverage, at low temperatures and with low amount of defects.

**Figure 1:**
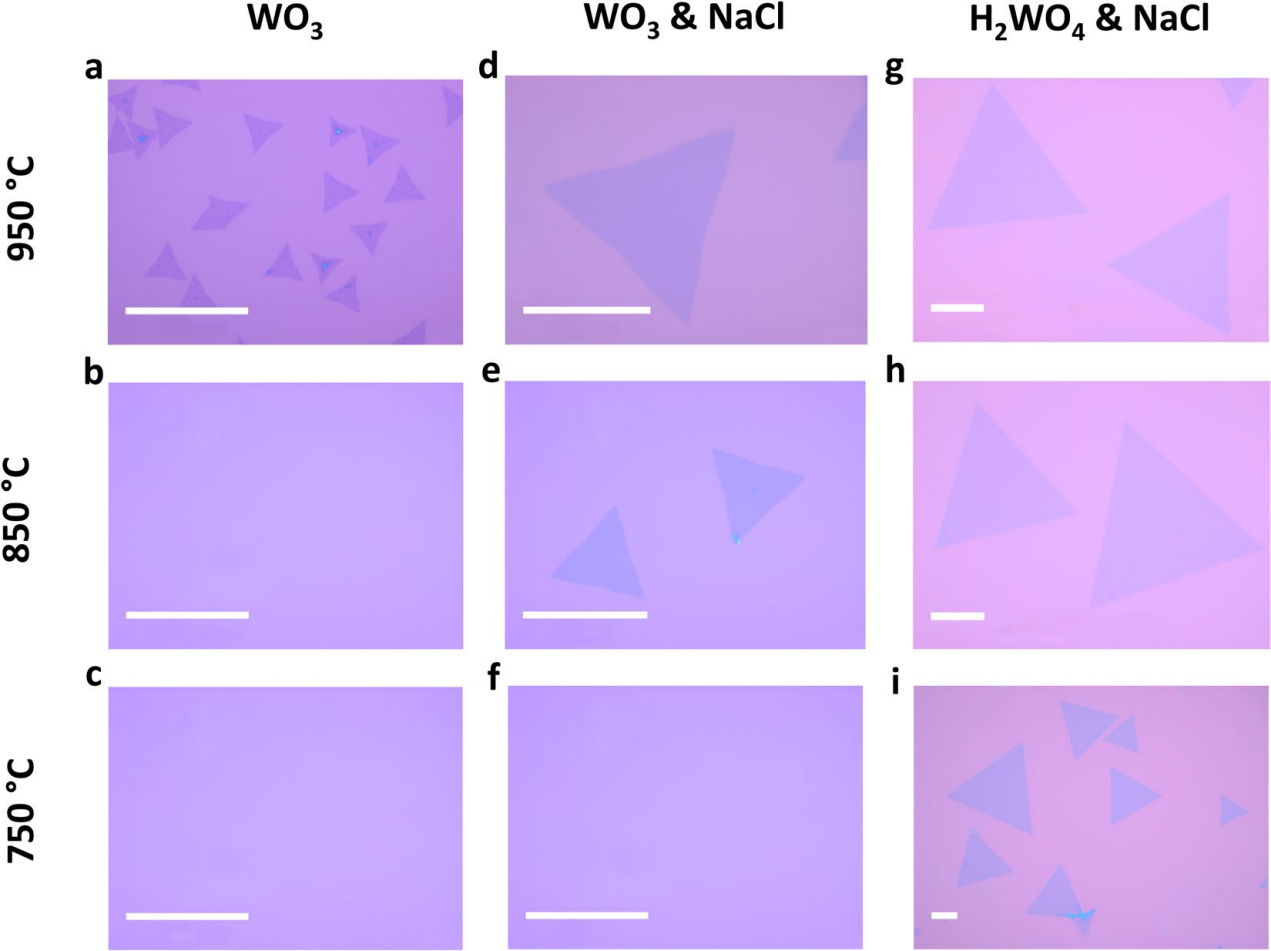
Optical micrographs of WS_2_ triangles grown on SiO_2_/Si substrates at different temperatures and using different precursors: (**a**) WO_3_ at 950 °C; (**b**) WO_3_ at 850 °C; (**c**) WO_3_ at 750 °C; (**d**) WO_3_ + NaCl at 950 °C; (**e**) WO_3_ + NaCl at 850 °C; (**f**) WO_3_ + NaCl at 750 °C; (g) H_2_WO_4_ + NaCl at 950 °C; (**h**) H_2_WO_4_ + NaCl at 850 °C; (**i**) H_2_WO_4_ + NaCl at 750 °C. Scale bar is 50 μm.

Optical micrographs of WS_2_ monolayers (Fig. 1) grown using different tungsten oxide precursors systems at different temperatures (950 °C, 850 °C and 750 °C) show distinctively increasing lateral size of the triangles and increasingly facilitated synthesis at low temperatures from WO_3_ to WO_3_-NaCl and H_2_WO_4_-NaCl. The possibility to grow WS_2_ from WO_3_ at temperatures not lower than 950 °C is notoriously attributed to the high sublimation temperature of the oxide^17,28–30^. In specific, the size of the WS_2_ triangles increases from ~10 μm, to 60 μm, upto 200 μm with areas of continuous polycrystalline monolayered coverage of ~0.8 mm (Figure S2). Further, the WS_2_ synthesis at temperatures as low as 750 °C was enabled only by the precursors system of H_2_WO_4_-NaCl with remarkable triangular size of ~100 μm. In addition, increasing the growth pressure (from ~1 mbar to 13 mbar) at 950 °C, bilayered WS_2_ flakes are preferentially formed (Figure S3) using the H_2_WO_4_-NaCl system.

It is worth noting that larger WS_2_ domains obtained at 950 °C, as compared to 850 and 750 °C, can be explained in the light of the Robinson & Robin model^31^. At high temperature the diffusivity of the adsorbed precursors on the SiO_2_ surface is favourable leading to the expansion of the existing domains. At the same time, desorption of absorbed species is higher than at lower temperatures, limiting the achievement of a supersaturation concentration and thus reducing the nucleation density.

From structural investigation of the reaction products in the different precursors systems, we could elucidate the role played by the water intercalated in the H_2_WO_4_ and how this can enable the favorable synthesis at low temperature and with low density of defects as compared to the WO_3_-based precursors. From X-ray diffraction (XRD) characterization (Supporting Information) it was possible to observe that the main products of the reactions between NaCl and either H_2_WO_4_ or WO_3_ are similar: Na_x_W_y_O_z_ and tungsten oxychlorides (WClO_4_ and WO_2_Cl_2_). However the reactions occur at significantly lower temperatures for H_2_WO_4_ compared to WO_3_. While Na_x_W_y_O_z_ species possesses a high evaporation temperature, as they are found in the crucible (Figure S5) at the end of the synthesis of WS_2_, the tungsten oxychlorides are volatile (Figures S4, S5) and they can possibly play a key role in promoting the synthesis of WS_2_. Indeed the system H_2_WO_4_-NaCl is likely to enable the formation of tungsten-oxyhalide species (i.e., WO_2_Cl_2_, WOCl_4_) at lower temperatures than WO_3_-NaCl, as NaCl dissociation is promoted by the H_2_O molecules gradually released by H_2_WO_4_ upon heating. On the bases of previous studies on the synthesis of WS_2_ bulk crystals, the formation of tungsten oxychlorides (WO_2_Cl_2_ and WOCl_4_) is indeed likely to occur with higher chances with respect to the formation of metal halides (e.g. WCl_6_)^32,33^. Tungsten oxychlorides (WO_2_Cl_2_ and WOCl_4_) can be volatile from 200 °C^34^ and they can be sulfidized in vapour phase forming a few-atom clusters of W-S which can deposit onto the target substrate as adatom species^35^ where they can form WS_2_ via a diffusion-desorption mediated mechanism of nucleation and growth. WOCl_4_ has been previously used^36^ as precursor for the synthesis of WS_2_ bulk films. Despite its strong tungsten-oxygen double bonds, WOCl_4_ proved to be an effective precursor with a clean decomposition pathway without formation of tungsten oxysulfides. We have verified that using this precursor is indeed possible to obtain WS_2_ at temperatures as low as 550 °C (Figure S6a). The key role played by the oxyhalide species becomes apparent if we try to grow WS_2_ by using H_2_WO_4_ as single precursor. As this decomposes to form WO_3_, only small WS_2_ domains are observed with PL characteristics similar to the WO_3_ precursor-led growth (Figure S6b).

**Figure 2:**
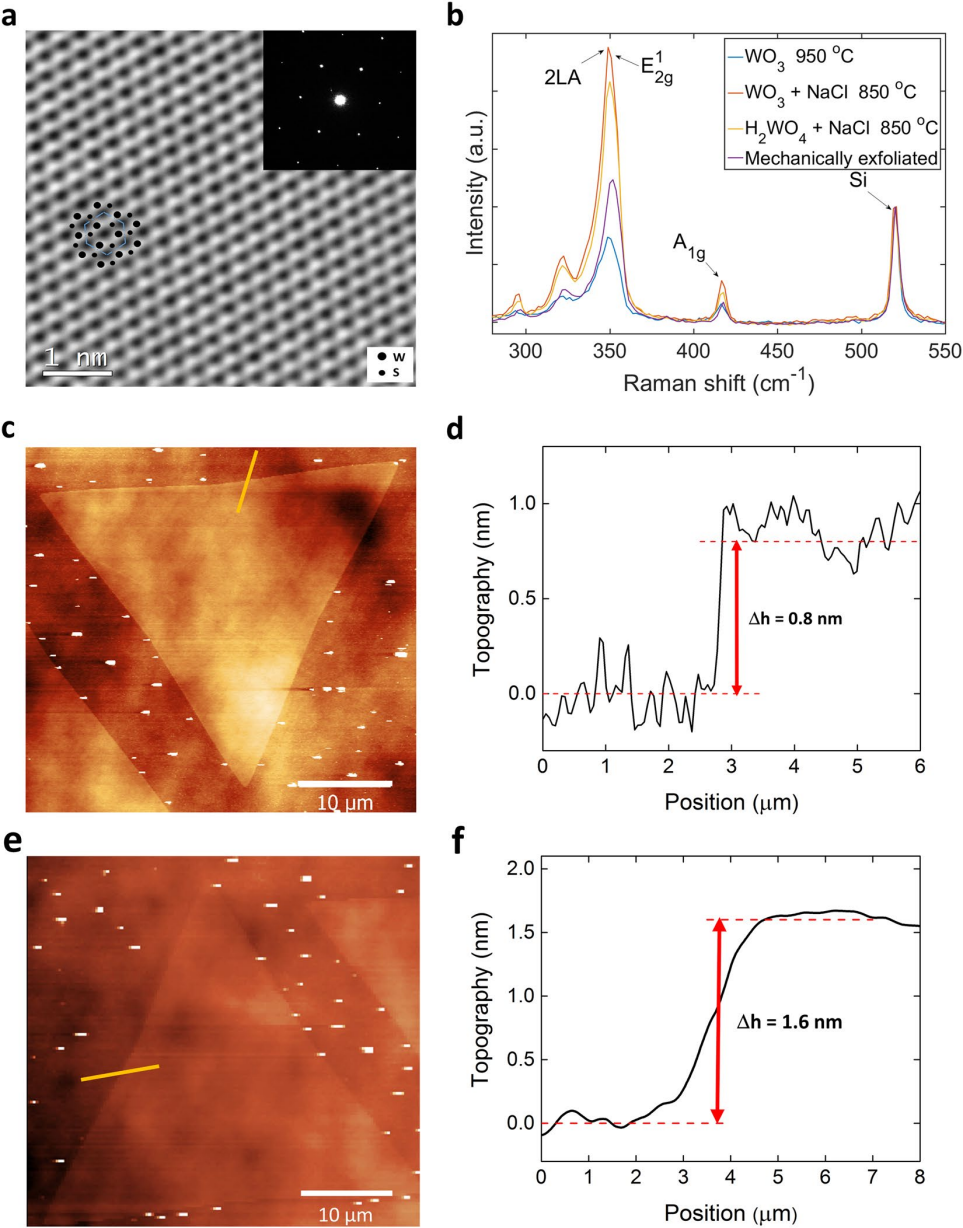
Structural and physical characterization of WS_2_ triangles: (**a**) Phase image of the reconstructed exit-plane wave function from a focal-series of HRTEM images of the WS_2_ lattice grown using H_2_WO_4_ + NaCl. The inset reports a selected diffraction area which shows an hexagonal pattern; (**b**) Raman spectra showing the characteristics active modes of WS_2_ grown under different conditions and compared with mechanically exfoliated flakes; (**c**) AFM image and (**d**) corresponding thickness profile of monolayer WS_2_; (**e**) AFM image and (**f**) corresponding thickness profile of bilayer WS_2_.

High-resolution transmission electron microscopy (HRTEM) imaging confirms the high crystalline nature of the material (Fig. 2a). The measured lattice constant is ~0.3 nm consistent with that of 2H-WS_2_ (a = 0.318 nm). The Raman spectra of WS_2_ obtained using the different precursors systems are shown in Fig. 2b. All of the spectra exhibit two characteristic peaks located at ~(351 ± 0.53) cm^−1^ and ~(417.6 ± 1) cm^−1^, which can be attributed to 2LA-E^1^_2g_ and A_1g_ Raman modes of pristine WS_2_ monolayer^37,38^. Interestingly, the distribution of the peak positions is increasingly narrower from WO_3_ precursor, WO_3_ + NaCl and to H_2_WO_4_ + NaCl (Figures S8, S10). The Raman peaks intensities are uniform across the entire triangle area (Figures S7, S9) and the frequency difference (Δν) between 2LA(M) and A_1g_ modes is ~(66.5 ± 0.53) cm^−1^ (Figure S11), as expected for monolayer WS_2_^37^. The AFM thickness profile analysis of WS_2_ triangles confirms the monolayer (Fig. 2c,d) and bilayer (Fig. 2e,f) nature of the flakes, showing an edge step height of ~0.8 nm (Fig. 2d) and ~1.6 nm (Fig. 2f), respectively^39,40^.

**Figure 3:**
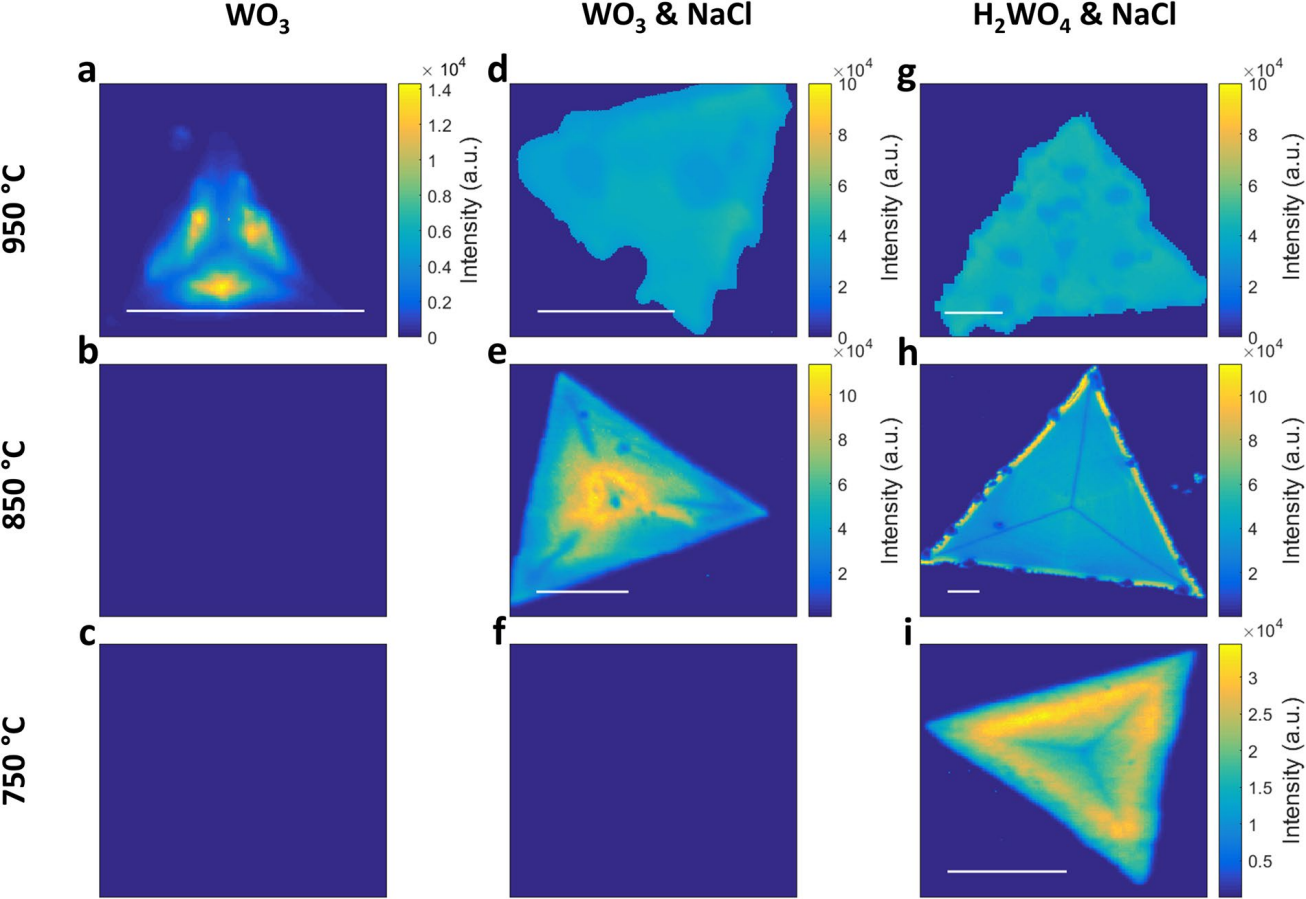
Spatial maps of PL intensity of WS_2_ grown in the conditions exemplified in Fig. 1. The scale bar length is 10 μm.

**Figure 4:**
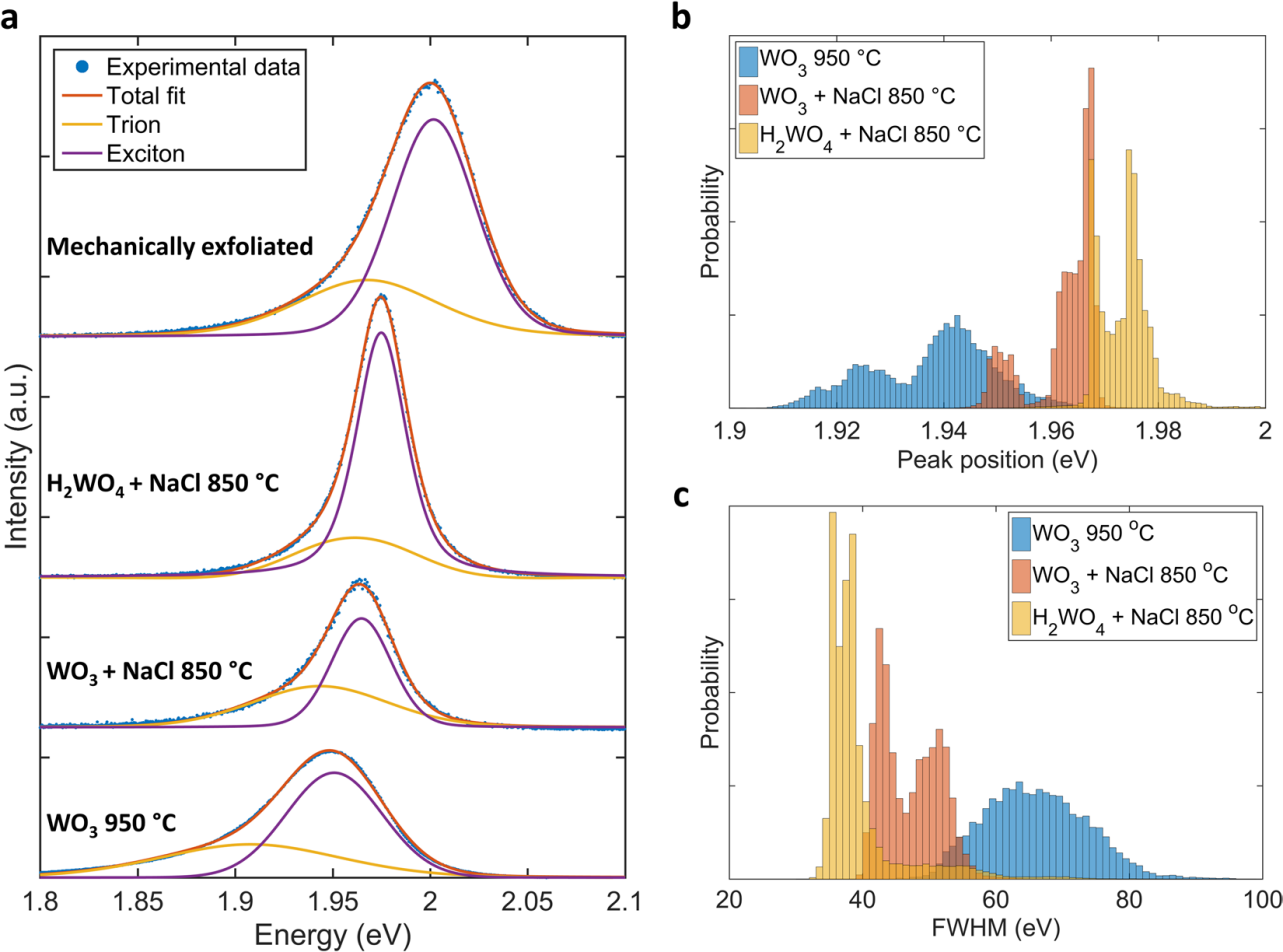
PL spectra characteristics of WS_2_ grown using: WO_3_ at 950 °C, WO_3_ + NaCl at 850 °C, H_2_WO_4_ + NaCl at 850 °C: (**a**) individual spectra (dotted line) and deconvolution in exciton and trion components; (**b**) distribution of PL peak position and (**c**) distribution of PL FWHM for several WS_2_ monolayers grown using the three different precursors systems.

A comparison of representative photoluminescence (PL) intensity maps of WS_2_ monolayers grown under the three precursors systems at different temperatures is reported in Fig. 3. The PL peak intensity appears consistently higher for the NaCl-based precursors system as compared to WO_3_. The intensity variation pattern across an individual flake is not yet fully understood^41^, and it is likely to be due to different defect concentrations in the form of sulfur vacancies. The FWHM of the PL peaks significantly decreases from ~75 meV, to ~50 meV, to ~36 meV for the three precursors systems, WO_3_, WO_3_ + NaCl, and  H_2_WO_4_ + NaCl respectively, with a distribution significantly narrower and more uniform across the same flake and different flakes (SI). It is worth noting that 36 meV of FHWM is narrower that mechanically exfoliated material (Fig. 4a) which is ~59 meV. This suggests that WS_2_ grown by using H_2_WO_4_ + NaCl possesses less structural defects compared to the other precursors systems, and specifically in the form of sulfur vacancies. Indeed, it has been reported that S vacancies increase the electron density, thus the trions population and the strength of the PL emission at lower energies than the optical band gap with a consequent increase of the FWHM^4,42^. The PL peak position progressively blueshifts from 1.94 eV, to 1.96 eV, to 1.98 eV, reaffirming a decreased amount of S vacancies and the formation of a progressively more pristine material.

**Figure 5:**
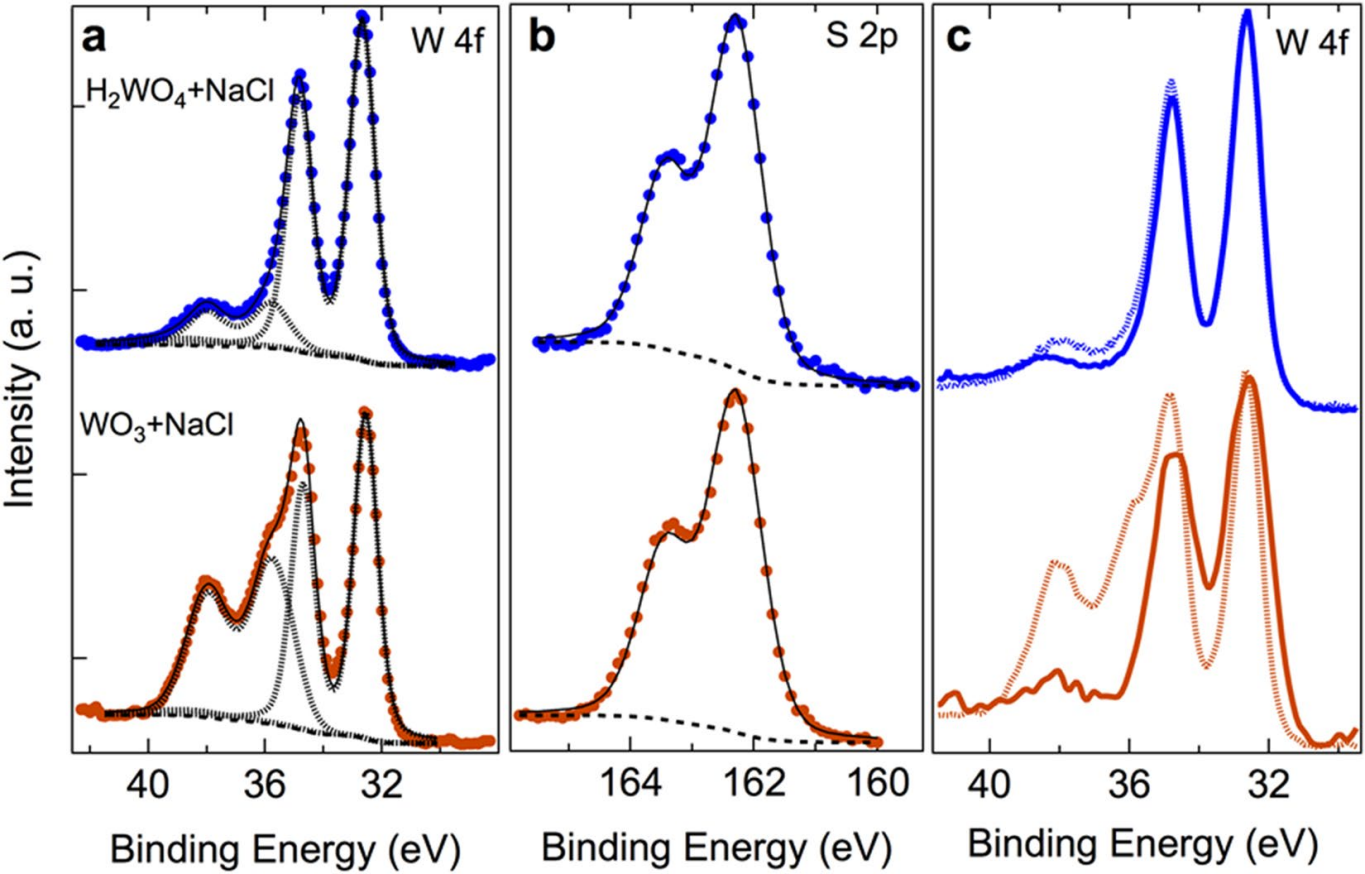
XPS spectra of the W 4 f and S 2p core level peak regions. (**a**) Comparison of W 4f_5/2_, W 4f_7/2_ and W 5p core levels of WS_2_ grown using H_2_WO_4_ + NaCl at 950 °C (blue spectrum) with WS_2_ grown using WO_3_ + NaCl at 950 °C (red spectrum). The deconvolution of W 4f_5/2_, W 4f_7/2_ and W 5p core levels and overall fit of the spectrum are reported as black dashed and a continuous line respectively. (**b**) The S 2p_1/2_ and 2p_3/2_ core levels for each of the two growth conditions are reported in the central panel. (**c**) W 4f_5/2_, W 4f_7/2_ and W 5p core levels before (dashed line) and after transfer (continuous like) onto a new SiO_2_/Si substrate are compared showing the complete disappearance of the residual WO_3_ components. The spectra were fit by Doniach-Sunjic function after subtracting a Shirley background (black dashed line).

A molecular conversion based-growth mechanism, were tungsten oxyhalide molecules are sulfidized in vapour phase, versus a topotactic-like conversion of WO_3_ in WS_2_ can explain the different defects contents in WS_2_. The greater efficiency of H_2_WO_4_ in inducing a complete sulfidization of the precursors compared with the WO_3_ + NaCl system has been also confirmed by chemical analysis (X-ray photoelectron spectroscopy). Analysing WS_2_ grown using H_2_WO_4_ + NaCl, the W 4f_5/2_ and W 4f_7/2_ core levels (Fig. 5a) present peak position characteristic of W^4+^ in WS_2_^43,44^ (32.7 and 34.8 eV respectively) and the narrowest achievable FWHM (1 eV) (Fig. 5a), using the Mg Kα as X-ray source. This indicates chemical purity and expected stoichiometric ratio of W and S. This has been also confirmed by calculating the concentration of S and W from the integrated intensity of the W 4 f and S 2p core levels. The S 2p_1/2_ and 2p_3/2_ core levels, also appear at the expected position for WS_2_ (162.3 eV and 163.4 eV respectively, Figure 5b)^44^ and with a very narrow FWHM (1 eV) (Fig. 5c). A very small amount of W^6+^ (W 4f_5/2_ and W 4f_7/2_ core levels centred at 35.9 eV and 38.1 eV respectively in Fig. 5a) attributable to WO_3_, which partially overlaps with the W 5p core level (38.5 eV), can be observed which however disappears after transferring the flakes on a new SiO_2_/Si substrate (Fig. 5c) and thus suggesting that it is related to residual precursors on the substrate considering the XPS spot size is ~1 mm. It is worth noting that after transfer the FWHM of the W 4 f core levels remains unchanged suggesting that the transfer process preserves the crystallinity of the flakes and no additional defects are introduced.

Similarly, chemical purity and expected stoichiometric ratio of 2:1 for S:W have been observed for WS_2_ grown from WO_3_ + NaCl (Fig. 5a). Nevertheless, a larger W^6+^ contribution, attributable to WO_3_ (W 4f_5/2_ and W 4f_7/2_ centred at 35.9 eV and 38.1 eV respectively in Fig. 5a) has been detected in this case suggesting that a conspicuous amount of precursors does not get sulfurized and it is just deposited onto the SiO_2_ wafer. Upon transfer on a new SiO_2_/Si substrate, this component entirely disappears (Fig. 5c), thus indicating also in this case that WO_3_ is mainly distributed on the substrate. The FWHM of the W^4+^ 4 f core levels is ~1.2 eV in this case, suggesting higher concentration of defects compared to H_2_WO_4_ + NaCl-led growth (Fig. 5a). The transferred WS_2_ present a FWHM even larger ~1.3 eV, suggesting the introduction of atomic defects as a consequence of the mechanical stress underwent by the flakes with preexisting defects (Fig. 5c). To conclude, XPS study confirms the effectiveness of H_2_WO_4_ as precursor versus WO_3_.

**Figure 6:**
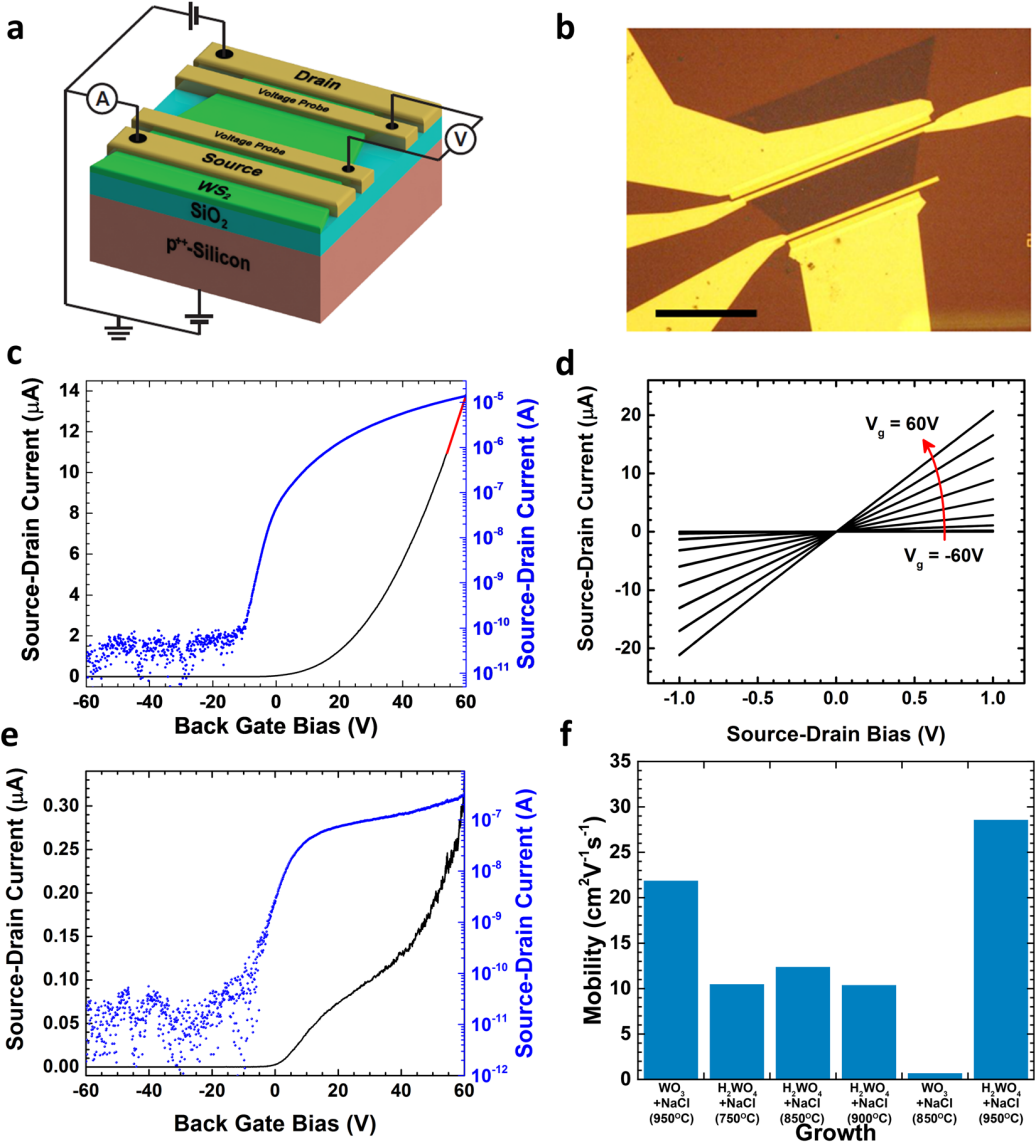
Electrical characteristics of monolayer WS_2_: (**a**) Schematic of the bottom-gated field effect transistors; (**b**) optical micrograph of the device (scale bar is 20 μm); (**c**) FET transfer curve for the monolayer WS_2_ grown using H_2_WO_4_ + NaCl at 950 °C showing the highest mobility of 28 cm^2^/Vs (linear region of the transport graph marked with a red-dashed line); (**d**) Response curves at different gate biases for a WS_2_ triangle grown using H_2_WO_4_ + NaCl; (**e**) FET transfer curve for the monolayer WS_2_ grown using WO_3_ + NaCl at 850 °C; (**f**) electron mobilities of monolayers WS_2_ grown using different conditions.

The progressive reduction of structural defects from WO_3_ to H_2_WO_4_ has been proven by electrical characterization. The electrical properties of the WS_2_ flakes were characterised through their performance in bottom-gated field effect transistors (FET) (Fig. 6a,b). The FET transfer curve (Fig. 6c) displays an accumulation-type *n*-channel transistor, where the current flowing through the channel increases with increased gate bias, after the threshold voltage.

**Figure 7:**
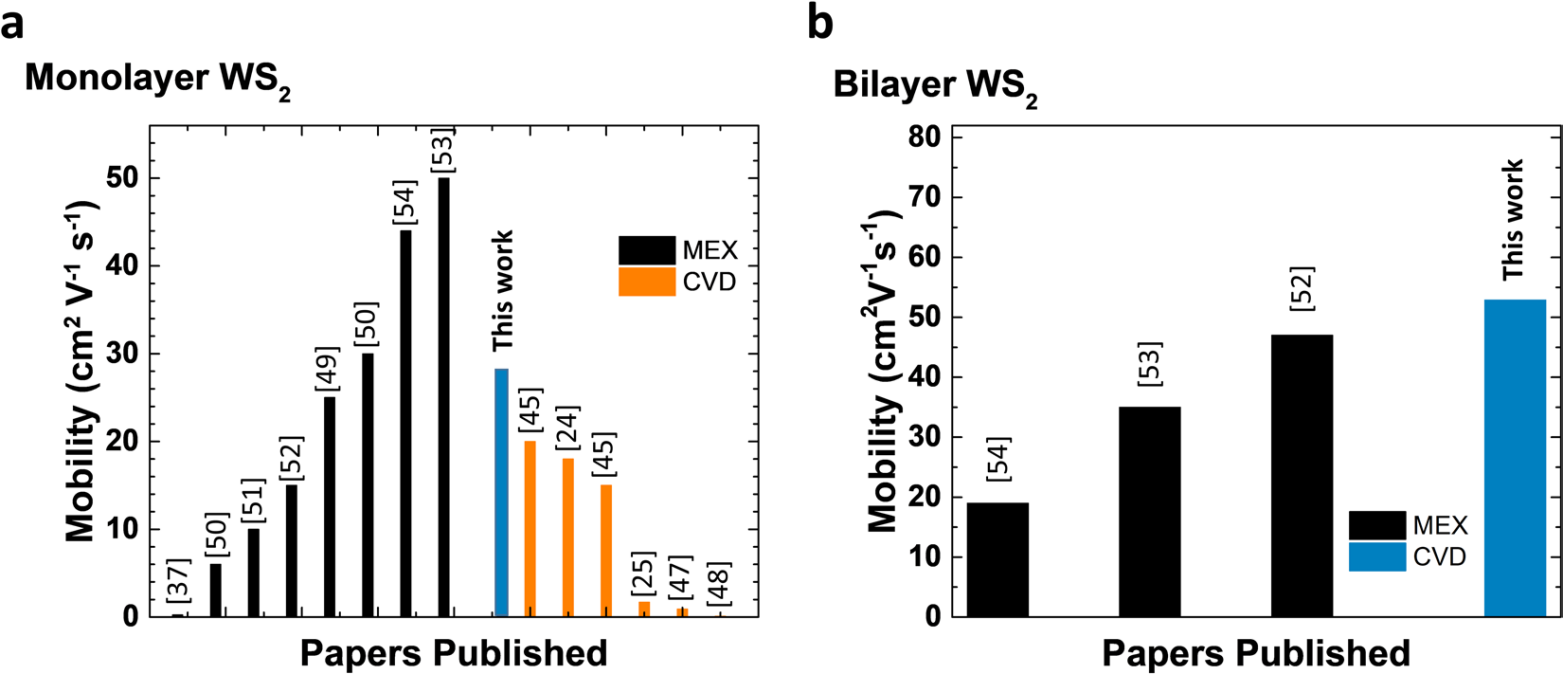
Comparison of our results with the literature of CVD grown material and mechanically exfoliated WS_2_ (MEX): electron mobility for (**a**) monolayer WS_2_ and (**b**) bilayer WS_2._ The histograms show our record values for both monolayer and bilayer amongst the best values reported for CVD grown WS_2_.

**Figure 8:**
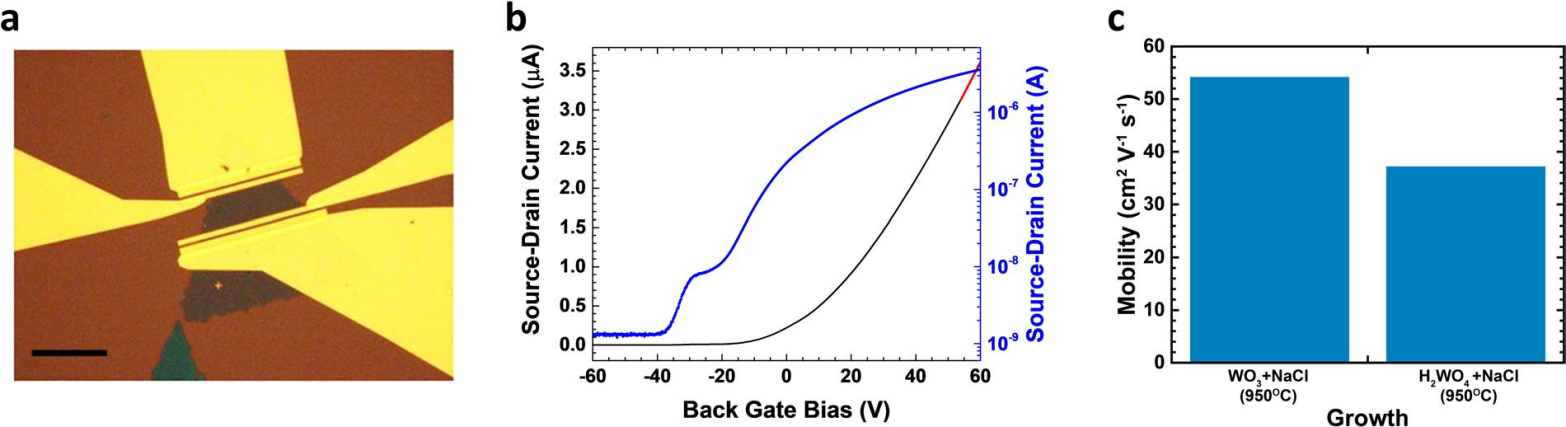
Electrical characteristics of bilayer WS_2_: (**a**) Optical micrograph of the device (scale bar is 30 µm); (**b**) FET transfer curve for the bilayer WS_2_ grown using WO_3_ + NaCl at 950 °C showing the highest mobility of 52 cm^2^/Vs (linear region of the transport graph marked with a red-dashed line); (**c**) electron mobility of bilayer WS_2_ grown by using different precursors systems.

The field-effect mobility was calculated in the linear region of the transport graph (marked with red-dashed line in Fig. 6c), using *µ*_n_ = *C*_ox_^−1^(d*σ*/d*V*_gs_). Overall, monolayer WS_2_ grown using H_2_WO_4_ + NaCl shows electron mobilities systematically higher compared with the WO_3_ + NaCl system (Fig. 6c,e,f) corroborating the fact that higher crystal quality is expected by using H_2_WO_4_ as precursor. Further, monolayer WS_2_ presents electron mobility of ~(28 ± 1.4) cm^2^/Vs (Fig. 6c,f) which is the highest mobility reported so far for CVD grown WS_2_ deposited onto SiO_2_ (Fig. 7a)^19,21,23–25,30,45–49^ and comparable to mechanically exfoliated WS_2_^37,50–52^. The highest mobilities using either H_2_WO_4_ + NaCl or WO_3_ + NaCl are displayed at 950 °C (Fig. 6f) suggesting that the growth temperature does also play a role in improving the crystal quality of the material. While the role played by the different precursors systems in determining the crystallinity of the synthesis product becomes more prominent at low growth temperatures. Monolayer WS_2_ grown using H_2_WO_4_ + NaCl exhibits electron mobilities of ~(10 ± 1) cm^2^/Vs at temperatures between 750 °C and 900 °C. While the electron mobilities of monolayer WS_2_ grown using WO_3_ + NaCl at 850 °C (Fig. 6f) present lower values of ~(0.4 ± 0.1) cm^2^/Vs. Bilayer WS_2_ shows electron mobility systematically higher than monolayer and also systematically higher than mechanically exfoliated bilayered flakes^52,53^ (Fig. 7b). The electron mobility of ~(52 ± 4) cm^2^/Vs (Figs 7 b, 8a,b,c) represents a record mobility as compared to CVD grown or mechanically exfoliated bilayer WS_2_ onto SiO_2_ reported so far^52–54^. The fact that the highest mobility for bilayer WS_2_ has been obtained using WO_3_ + NaCl with no use of H_2_WO_4_ suggests that the bilayer system is less affected by the precursos choice, and a bilayered material presents in general crystal quality superior to monolayers.

## Conclusions

In conclusion, we have developed a synthesis strategy which enables high crystal quality WS_2_ as reflected in the high optical quality and in the carrier mobility that overcome naturally occurring materials. The molecular precursors approach leads to effective sulfidization of W, revealing to be highly advantageous with respect to the traditional oxide–based conversion synthesis of WS_2_. These results can be translated and applied to the synthesis of different TMDCs, and pave the way towards industrially scalable synthesis of monolayer WS_2_ over large areas.

## Methods

### CVD Synthesis of WS_2_

Commercial WO_3_ (0.3 g, 99.9%, Sigma Aldrich), H_2_WO_4_ (0.3 g, 99.9%, Sigma Aldrich) and NaCl (0.3 g, ≥99.5%, Sigma Aldrich) powders were loaded in an alumina boat placed in the center of a 2 inch-diameter horizontal quartz tube CVD furnace. While an alumina boat containing sulfur powders (0.6 g, ≥99.5%, Sigma Aldrich) was loaded in the upstream zone of the tube, whose temperature was independently controlled by a different heater. The growth substrates were Si wafers (500 microns) with on top 285 nm of SiO_2_ thermally deposited. The substrates were sequentially cleaned for 15 min in acetone, isopropanol and deionized water in a sonicator, followed by dipping in H_2_SO_4_/H_2_O_2_ (3:1) for two hours and rising in deionized water. They were then blow dried with nitrogen gas, cleaned with O_2_ plasma for 5 min and loaded into the downstream zone of the furnace. The CVD growths were then performed at low pressure (~10^−1^ mbar) and under flow of high purity Ar gas (flow rate of 100 sccm). The furnace was heated to 750–950 °C with a ramp rate of 25 °C min^−1^, kept at the growth temperatures for 15 min and then naturally cooled down to room temperature. The sulfur powder was independently heated to 125 °C with a ramp of 5 °C min^−1^, kept at this temperature for 15 min and naturally cooled down.

### Sample Transfer

The transfer procedure was performed by depositing a PMMA film 350 nm thick onto the target sample, which was subsequently immersed in a KOH solution (0.1 M) until detachment of the PMMA from the SiO_2_/Si substrate. The PMMA/WS_2_ films was then scooped out with a new Si/SiO_2_ substrate, repeatedly washed in deionized water and then immerged in an acetone bath at 45 °C for 20 min to dissolve the PMMA film.

### TEM Characterization

TEM analysis of the WS_2_ flakes was carried out on a FEI Titan 80–300 S/TEM operated at 80 kV, equipped with a monochromator and a Cs aberration image corrector. Focal series micrographs of the representative flake were acquired at different objective lens focus values (using a spherical aberration coefficient Cs ~ −4 µm) and exit-wave reconstruction was performed using TrueImage software (FEI).

### Physical Characterization

Raman and photoluminescence spectra were collected using a Renishaw inVia spectrometer equipped with a 532 nm laser excitation. All the spatial maps were collected under a 100x objective using grating of 1800 line/mm, which provide a resolution of ~1.5 cm^−1^.

### X-ray Photoelectron Spectroscopy

X-Ray photoemission spectra were acquired in a custom made ultra-high vacuum system (pressure < 10^−9^ mbar) equipped with a VG Escalab Mk-II electron analyzer and a twin anode (Al/Mg) non-monochromatized x-ray source (Omicron DAR 400). All measurements were taken in quasi-normal emission (5° off) at room temperature using a pass-energy of 20 eV, an energy step of 0.1 eV and the Mg K_α_ emission line as exciting radiation.

### Device Fabrication

For the field-effect mobility measurements, single and bilayer WS_2_ field-effect transistors (FET) were fabricated after transfer to a new Si/SiO_2_ substrate where the Si is highly *p*-doped and acts as a global gate electrode, and a 285 nm thick thermally grown SiO_2_ serves as the gate dielectric. The FETs were fabricated on dried Si/SiO_2_/WS_2_ samples. Besides the current bearing contacts, termed here “Source” and “Drain”, two additional voltage probes were added to each FETs to allow for an accurate determination of the channel’s conductance by eliminating the contribution of the contacts. Both the current bearing leads and the voltage probes were patterned simultaneously using a standard electron beam lithography process. The source, drain and voltage probes consisted of 50 nm Au, while the electronics leads consisted of 5 nm Ti and 50 nm Au. A two-steps annealing process followed the fabrication. The samples were first annealed for 2 hours at 200 °C under H_2_/Ar (10/90) flow in atmospheric pressure, to etch residues of PMMA that was used as an electron resist for the lithography step. Then, the samples were put under a high vacuum (~10^−6^ mbar) and baked at 115 °C for 60 hours, to promote desorption of water molecules from the channel surface.

### Electrical Measurements

The FETs were measured inside the vacuum chamber at a constant pressure of ~10^−6^ mbar, without exposure to ambient conditions after the second annealing step. The drain electrode was biased with a low noise source-meter and the source electrode was grounded throughout the experiment. An additional source-meter was used to bias the global gate electrode, with respect to the source. The transistor current, *I*_ds_, was measured using an ammeter and the potential difference across the voltage probes, *V*_A-B_, was measured with a voltmeter. The channel conductivity, σ, is then readily obtained using σ = (*L I*_ds_)/(*W V*_A-B_), where *W* and *L* are the channel’s width and length, respectively. The measurement set-up is shown schematically (not to scale) in Fig. 6a. The oxide capacitance was estimated to be 115 µFm^−2^ from *C*_ox_ = ε_0_ ε _r_/*d*_ox_, where *d*_ox_ is the oxide thickness and ε_0_ and ε_r_ are the vacuum permittivity and SiO_2_ relative permittivity, respectively.

### Data availability statement

All relevant data generated or analysed during this study are included in this article and its Supplementary Information file.

## Electronic supplementary material

Supporting Information
