# Supplementary material for: High-Mobility and High-Optical Quality Atomically Thin WS2
Source: Sci Rep. 2017 Nov 2;7:14911. doi: 10.1038/s41598-017-14928-2 (PMC5668258; doi:10.1038/s41598-017-14928-2)
Supplement: Supplementary file 1 — Supporting Information [file 41598_2017_14928_MOESM1_ESM.pdf]

Supporting Information

“High-mobility and high-optical quality atomically thin WS_2”

Francesco Reale[a], Pawel Palczynski[a], Iddo Amit[b], Gareth F. Jones[b], Jake D. Mehew[b], Agnes Bacon[b], Na Ni[a], Peter C. Sherrell[a], Stefano Agnoli[c], Monica F. Craciun[d], Saverio Russo[b], Cecilia Mattevi*[a]

[a] Department of Materials, Imperial College London, SW7 2AZ, UK

[b] Centre for Graphene Science, Department of Physics, University of Exeter, Stocker Road, Exeter, EX4 4QL, UK

[c] Department of Chemical Sciences, University of Padua, Via F. Marzolo 1, 35131 Padua, Italy

[d] Centre for Graphene Science, Department of Engineering, University of Exeter, North Park Road, Exeter, EX4 4QF, UK

*c.mattevi@imperial.ac.uk

CVD synthesis of mono- and bi-layer WS_2

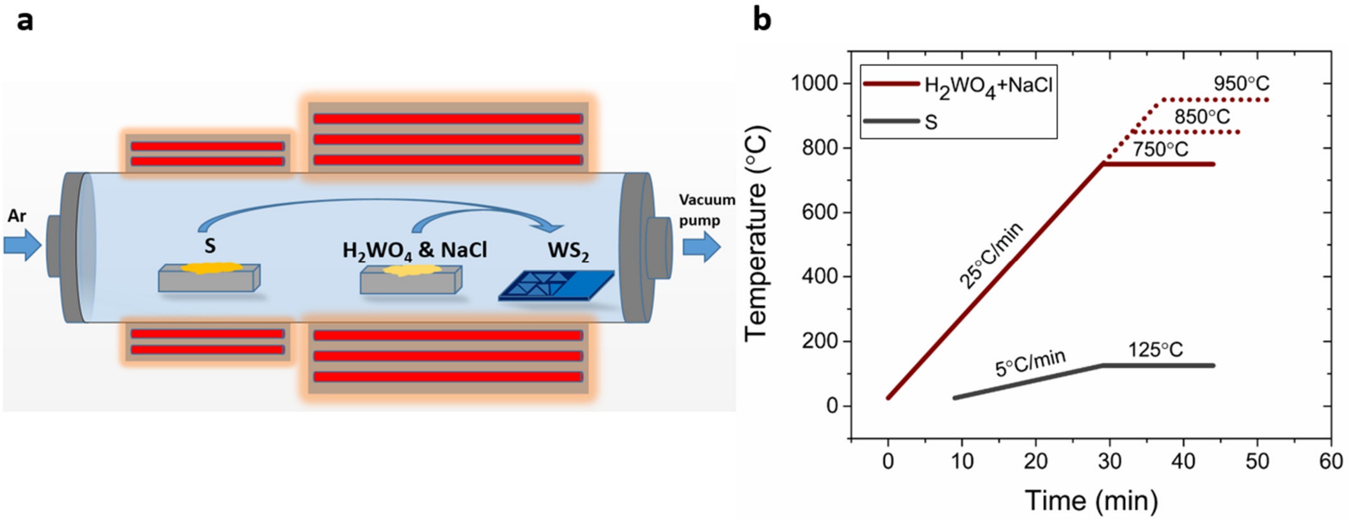

Figure S1. *Illustration of: (a) CVD tubular furnace set-up; (b) temperature profile of the sulphur and the W-precursors heaters respectively. The sulphur reaches 125 °C when the metal precursors are at the maximum temperature. The SiO_2/Si wafers used as substrate for WS_2 growth are placed 1-8 cm downstream the W-precursors crucible and they are subjected to the same temperature.*

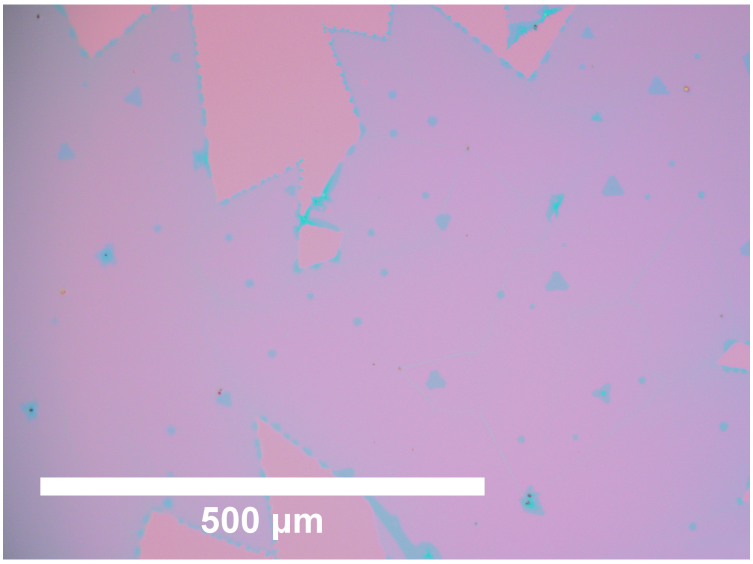

Figure S2. *Optical micrograph of a continuous polycrystalline WS_2 monolayer coverage.*

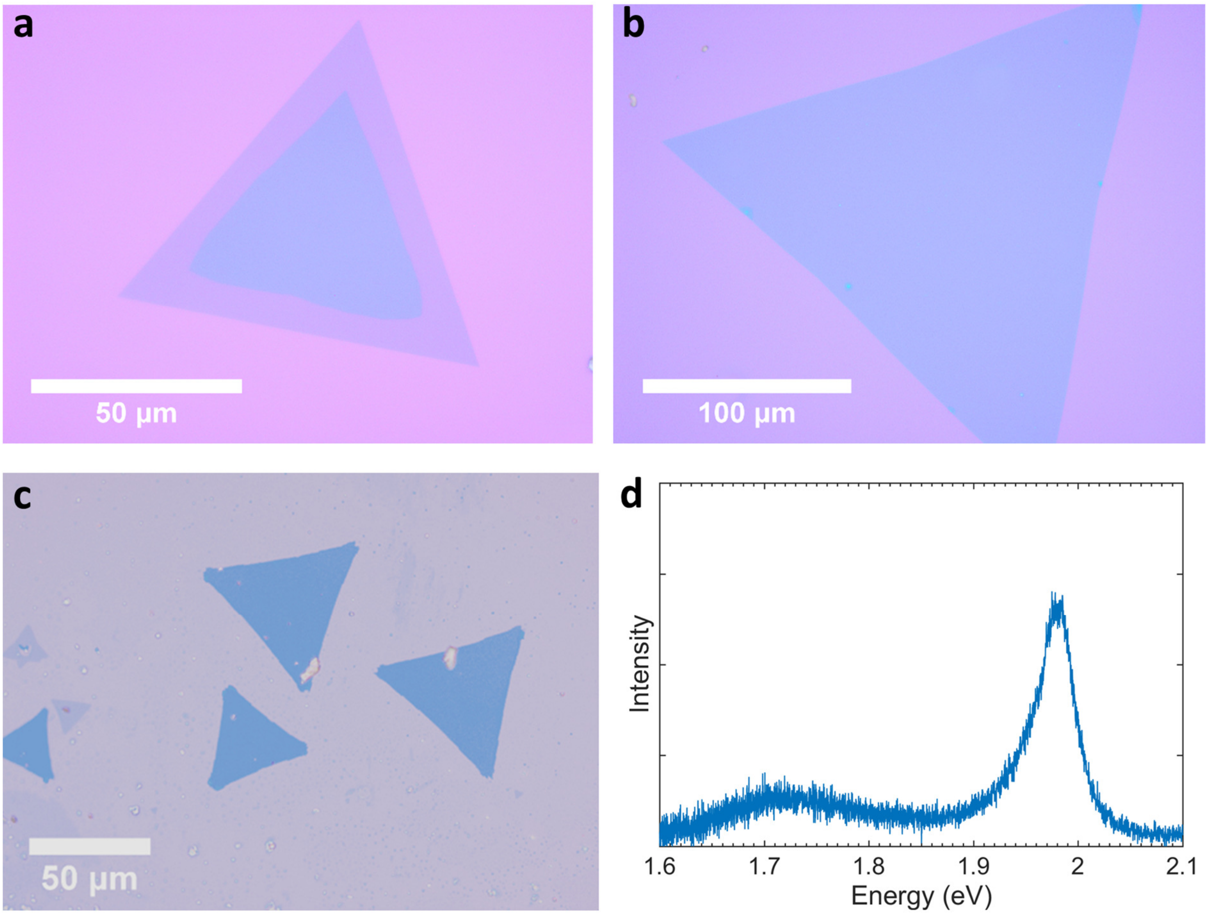

Figure S3. *Optical micrographs of (a) second WS_2 layer growing on top of a monolayer WS_2 triangle, (b) uniform bilayer WS_2 triangle, (c) bilayer WS_2 flakes with high nucleation density. (d) PL spectrum of a bilayer WS_2.*

Reaction mechanism

We found that the reaction between H_2WO_4 or WO_3 with NaCl (Figure S4) leads to the formation of $Na_xW_yO_z$ compounds (i.e., $Na_2W_4O_{13}$, $Na_2W_6O_{19}$), however the reaction occurs at significantly lower temperatures (~650 °C) for H_2WO_4 compared to WO_3 (~750°C). This means that the system H_2WO_4-NaCl releases Cl from 650 °C enabling formation of volatile tungsten-based oxyhalide species (i.e., WO_2Cl_2, $WOCl_4$)[1] at lower temperatures. NaCl dissociation is promoted by the H_2O molecules gradually released by H_2WO_4 upon heating. Indeed, the solid state structure of H_2WO_4 consists of vertex-sharing layers of octahedrally coordinated $WO_3(H_2O)$ units[2]. The layers are bonded together by hydrogen bonds between the terminal oxygen atoms and coordinated water molecules in neighbouring layers[3]. Thus, the weakly bonded H_2O molecules can be liberated at temperatures as low as 100-200 °C and they can therefore decompose NaCl. In a system without water, the NaCl dissociation would occur at temperatures greater than 1000 °C. The facilitated growth of WS_2 by using the WO_3-NaCl system[4] can be explained with the inevitable presence of water impurities in the CVD reaction chamber, in particular when the synthesis is performed at atmospheric pressure. On the bases of the XRD analysis and literature data[2-11] four reaction steps have been identified as likely to occur in our water-assisted-halide-mediated growth of WS_2:

1) $H_2WO_4\textit{(s)} \rightarrow WO_3\textit{(s)} + H_2O\textit{(l)}$

2) $NaCl\textit{(s)} + H_2O\textit{(l)} \rightarrow Na^+\textit{(aq)} + Cl^-\textit{(aq)} + H_2O\textit{(l)}$

3) $6Na^+\textit{(aq)} + 6Cl^-\textit{(aq)} + 14WO_3\textit{(s)} \rightarrow Na_2W_4O_{13}\textit{(s)} + Na_2W_6O_{19}\textit{(s)} + [Na_2W_2O_7\textit{(s)}] + WOCl_4\textit{(g)} + WO_2Cl_2\textit{(g)}$

4) $WOCl_4\textit{(g)} + WO_2Cl_2\textit{(g)} + S\textit{(g)} + H_2O\textit{(g)} \rightarrow WS_2\textit{(s)} + SO_2\textit{(g)} + H_2SO_3\textit{(g)} + HCl\textit{(g)} + S\textit{(g)}$

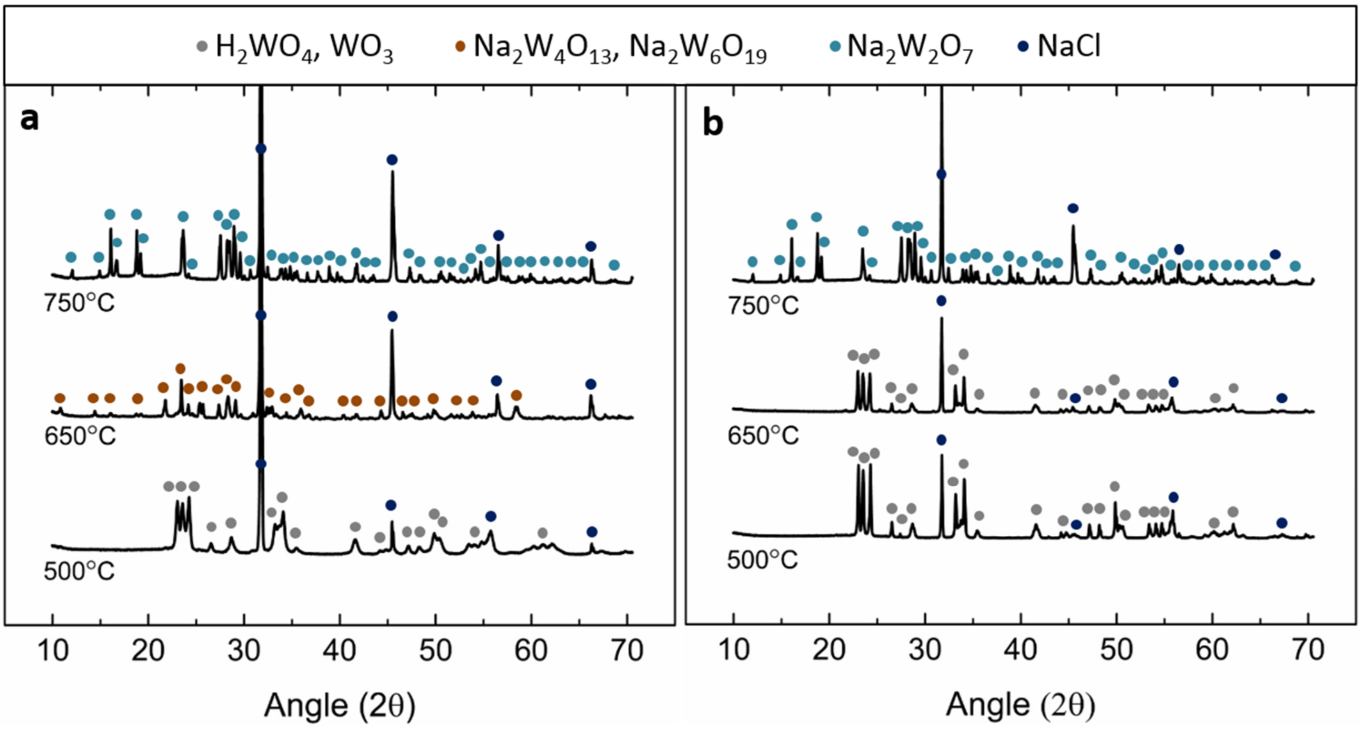

Figure S4. *XRD pattern of the residual powder of W-precursors after thermal treatment at 500 °C, 650 °C and 750 °C respectivley, using (a) H_2WO_4+NaCl and (b) WO_3+NaCl as precursor.*

Further, the formation of oxide-chloride species is also supported by the evidence that formation of tungsten oxyhalide species (WO_2X_2, WOX_4) has been reported for the synthesis of WS_2 bulk crystals via chemical vapor transport[12,13] by reacting halogen molecules such as Cl_2, I_2 and Br_2 used as transport agents and tungsten oxides. Further, the high volatility of the H_2WO_4+NaCl has been also confirmed by gravimetric analysis after growth. Growth performed by using WO_3 and H_2WO_4 only (Figure S5a,b) leads to a larger amount of WO_3 and substoichiometric WO_3 remaining in the crucible. On the contrary, WO_3 and H_2WO_4 mixed with NaCl undergo a significant weight loss and only a little amount of $Na_xW_yO_z$ (and unreacted NaCl) compounds is found (Figure S5c,d). In the case of H_2WO_4+NaCl, the remaining precursors is less than for the WO_3+NaCl growths. Furthermore, to confirm the key role played by Cl, we replaced NaCl with KCl and comparable growth results were obtained (Figure S6c).

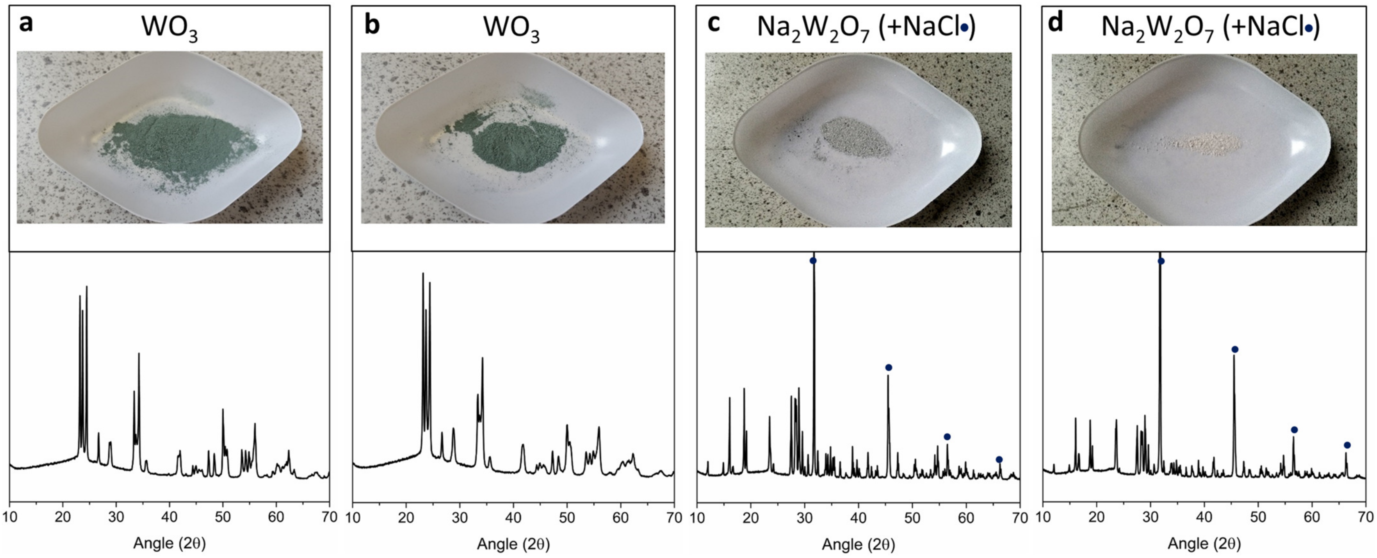

Figure S5. *Residual powders of the W-precursors and their XRD patterns after thermal treatment at 750°C using (a) WO_3, (b) H_2WO_4, (c) WO_3+NaCl, and (d) H_2WO_4+NaCl as precursor. The blue dots in the XRD patterns indicate the presence of residual NaCl.*

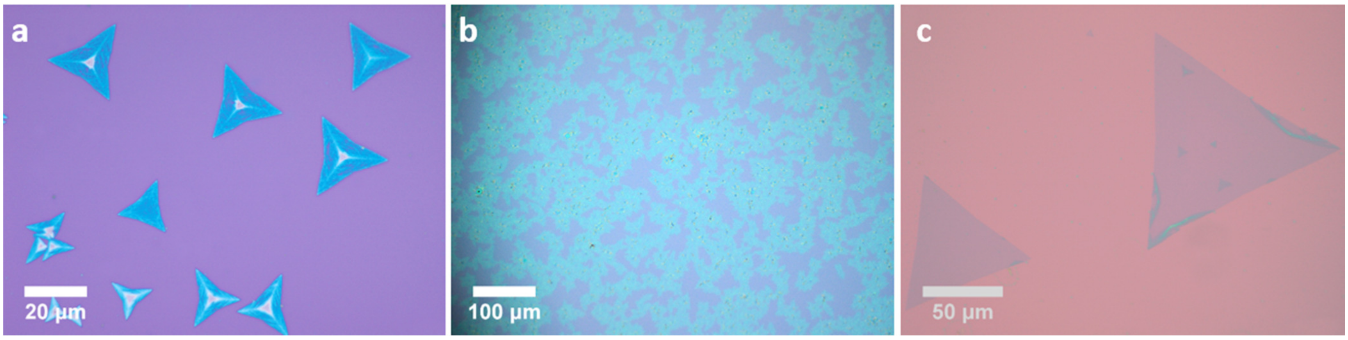

Figure S6. *Optical micrographs of WS_2 triangles and amorphous domains grown on SiO_2/Si substrates by using (a) $WOCl_4$ at 600°C, (b) only H_2WO_4 at 850°C, (c) H_2WO_4+KCl at 850°C.*

Raman Maps

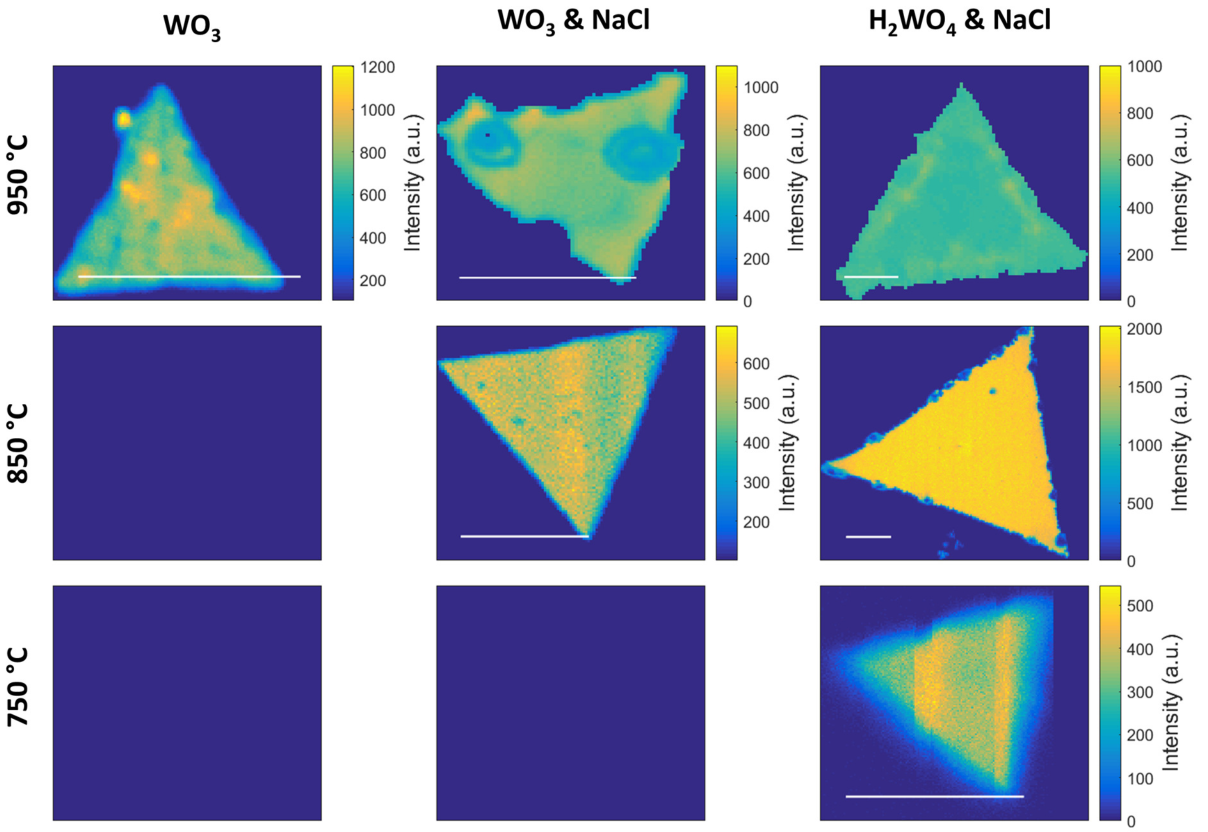

Figure S7. *Raman spectroscopy: 2LA+E^1_{2g} peak intensity. Scale bar 10 μm.*

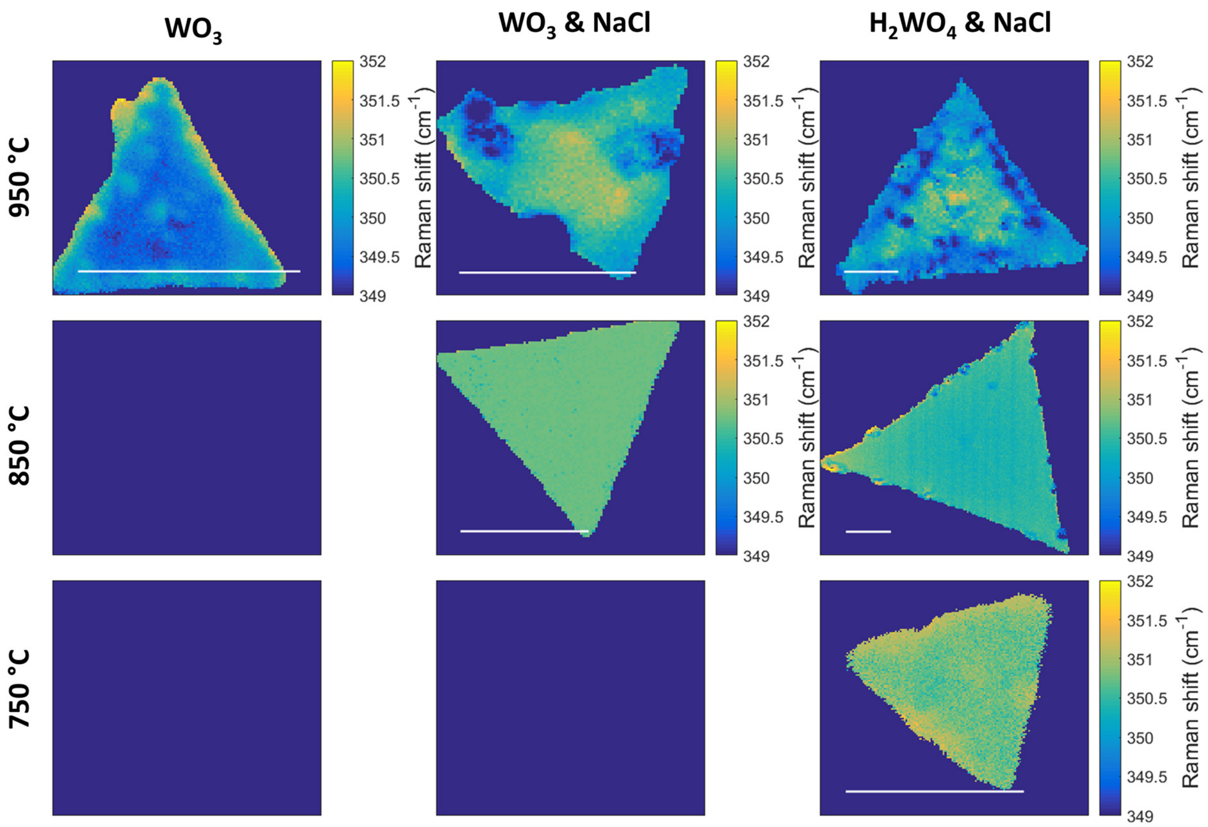

Figure S8. *Raman spectroscopy: 2LA+E^1_{2g} peak position. Scale bar is 10 μm.*

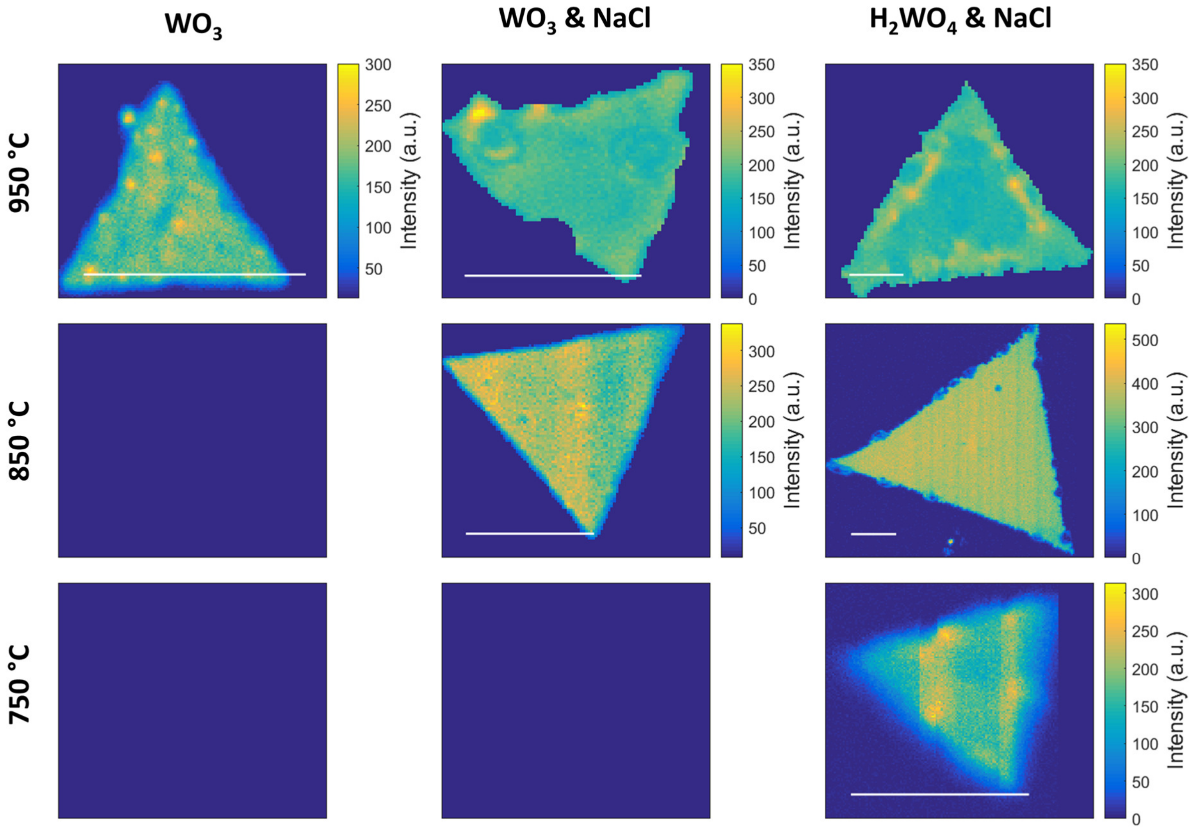

Figure S9. *Raman spectroscopy: A_{1g} peak intensity. Scale bar is 10 µm.*

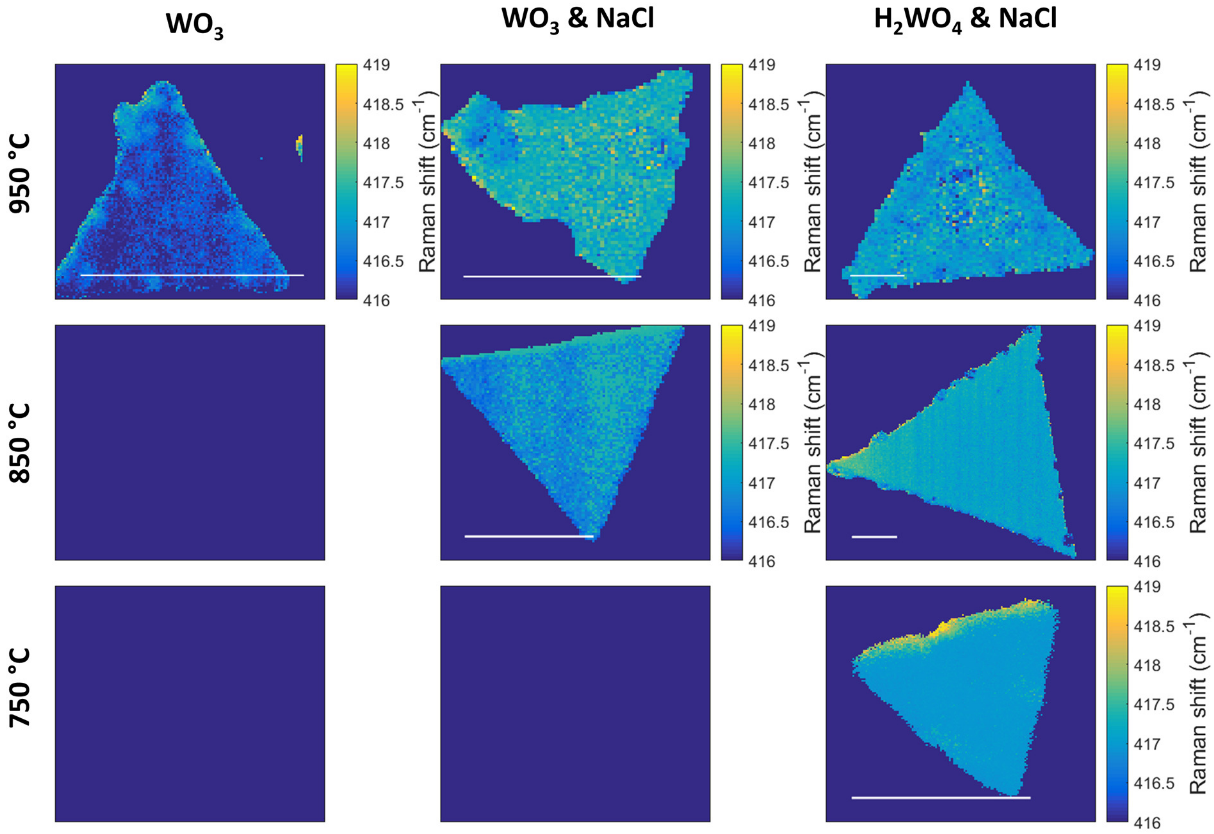

Figure S10. *Raman spectroscopy: A_{1g} peak position. Scale bar is 10 µm.*

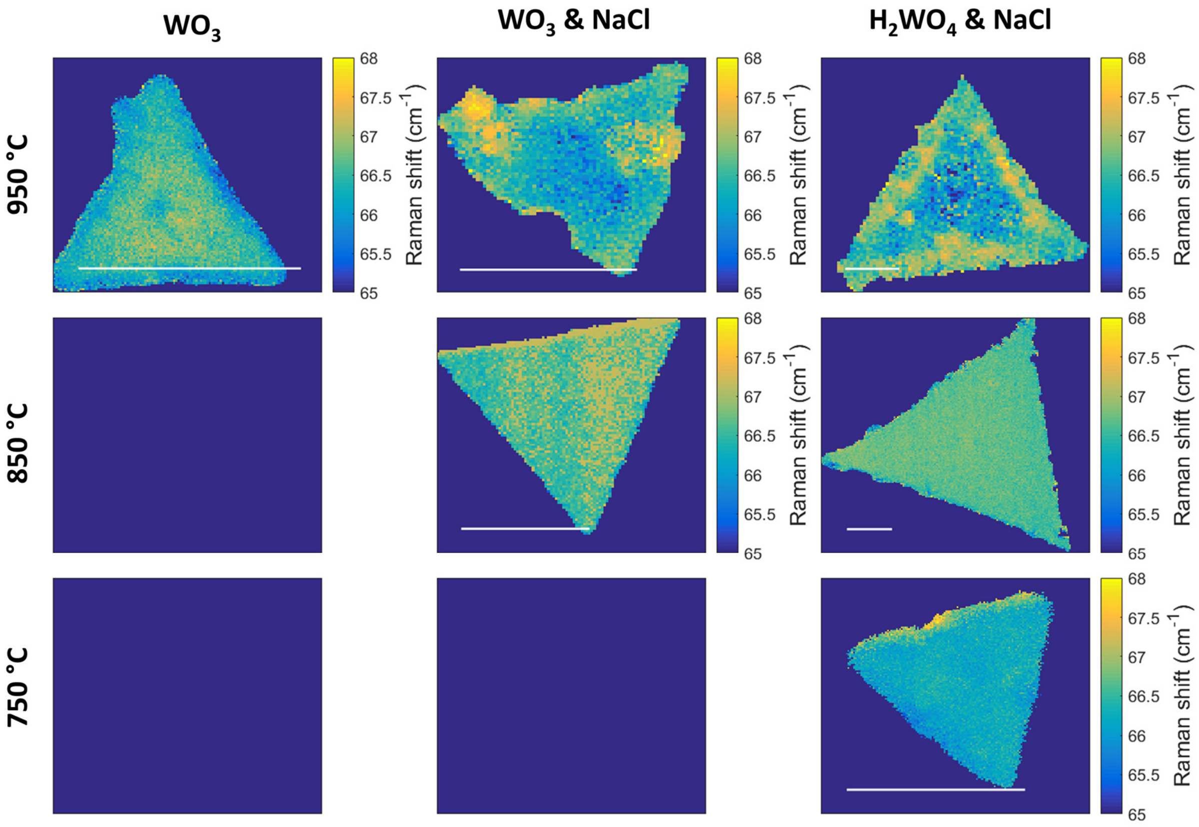

Figure S11. *Raman spectroscopy: 2LA-A_{1g} energy differences. Scale bar is 10 µm.*

Photoluminescence (PL) Characterization

The PL intensity map of WS_2 grown from WO_3 at 950 °C (Figure 3a) shows a three-lobes intensity pattern. The individual PL spectra appear asymmetric and they can be deconvolved in two distinct peaks, which have been attributed due to excitons and trions recombination (Figure 4)[14]. The PL peak positions over an individual WS_2 flake show a bimodal distribution, with high PL intensity distributed over three lobes characterized by a peak position distribution centred at 1.94 eV versus areas with lower intensity and peak position centred at 1.96 eV, with FWHM of ~75 meV and ~55 meV respectively (Figure 4, S12, S13, S15). Peak position and FWHM are comparable with the current state-of-the-art of WS_2 synthesized from WO_3[15-18]. The red shift of ~0.02 eV of the peak position (Figure 4b, S12) and

the large FWHM (Figure 4c, S13) of PL peaks form the three-lobe areas suggest higher concentration of structural defects[19,22]. These can be in the form of S-vacancies that increase the electron density, consequently the trions population[15,20] and strength PL emission at lower energies than the optical band gap (Figure 4) causing an increased FWHM[15,20]. Strain variations in the lattice can also affect the light emission intensity and wavelength[21,22]. To prove whether lattice strain could affect the PL peak position and the intensity pattern, we have relaxed the lattice by cutting the WS_2 domain using a 532 nm laser. As reported in Figure S14, the PL intensity and peak position remain unchanged along with the Raman peak position. Thus, structural defects in variable concentration are likely to be the main responsible for the spatial variation of PL[23]. With the addition of NaCl, WS_2 can be grown at temperatures as low as 850 °C with good PL intensity (Figure 3d,e). The light emission occurs at higher energy and the FWHM is narrower as compared to the WO_3-led growth (Figure 4). Further, the PL peak position and FWHM distributions are narrower within the same triangle compared to the WO_3-led growth and although they are still broad across several WS_2 monolayers, the standard deviations (Figure 4, S12, S13, S16) are smaller. The light emitted has higher energy (~1.95±0.002 eV and ~1.96±0.002 eV) and the FWHM is narrower (~43±2.8 meV and ~51±3 meV) (Figure 4) compared to monolayer WS_2 synthesized from WO_3 and most of the reported works[15-18]. This indicates that the trions component is now significantly reduced suggesting that WS_2 grown from NaCl+WO_3 is affected by less structural defects as compared to WO_3 precursor. A molecular conversion based-growth mechanism, were tungsten oxyhalide molecules are sulfidized in vapour phase, versus a topotactic conversion of WO_3 in WS_2 can explain the different defects contents in WS_2 (Figure 3d, e).

The PL peak intensity maps of WS_2 grown using H_2WO_4+NaCl are reported in Figure 3g, h, i. It is possible to notice a good PL intensity from 750 to 950 °C. The FWHM is significantly narrower (~36±3 meV), and the PL energy (~1.980±0.005 eV) is higher compared to what observed for WO_3 and WO_3+NaCl precursors systems (Figure 4, S12, S13, S17). This suggests that WS_2 grown by using H_2WO_4+NaCl possess even less structural defects, and specifically in the form of sulfur vacancies, which lead to a negligible contribution from trions to the PL peak (Figure 4a). Further, no appreciable difference in FWHM is observed from flake to flake (Figure 4b,c), confirming the high reproducibility of the synthesis. The FWHM is smaller than previously reported values for CVD-grown[15-18] and

exfoliated WS_2[24,25] (Figure 4a) while it is comparable to WS_2 grown on van der Waals substrates[26] and to high quality exfoliated WS_2[27]. Overall the distributions of the PL peak position and the FWHM across several WS_2 monolayers are much narrower (5 meV and 3 meV respectively) (Figure S18) compared to the other precursors systems. Different intensity patterns across individual flakes can still be recognized, however, they present smaller intensity difference compare to the other growth conditions.

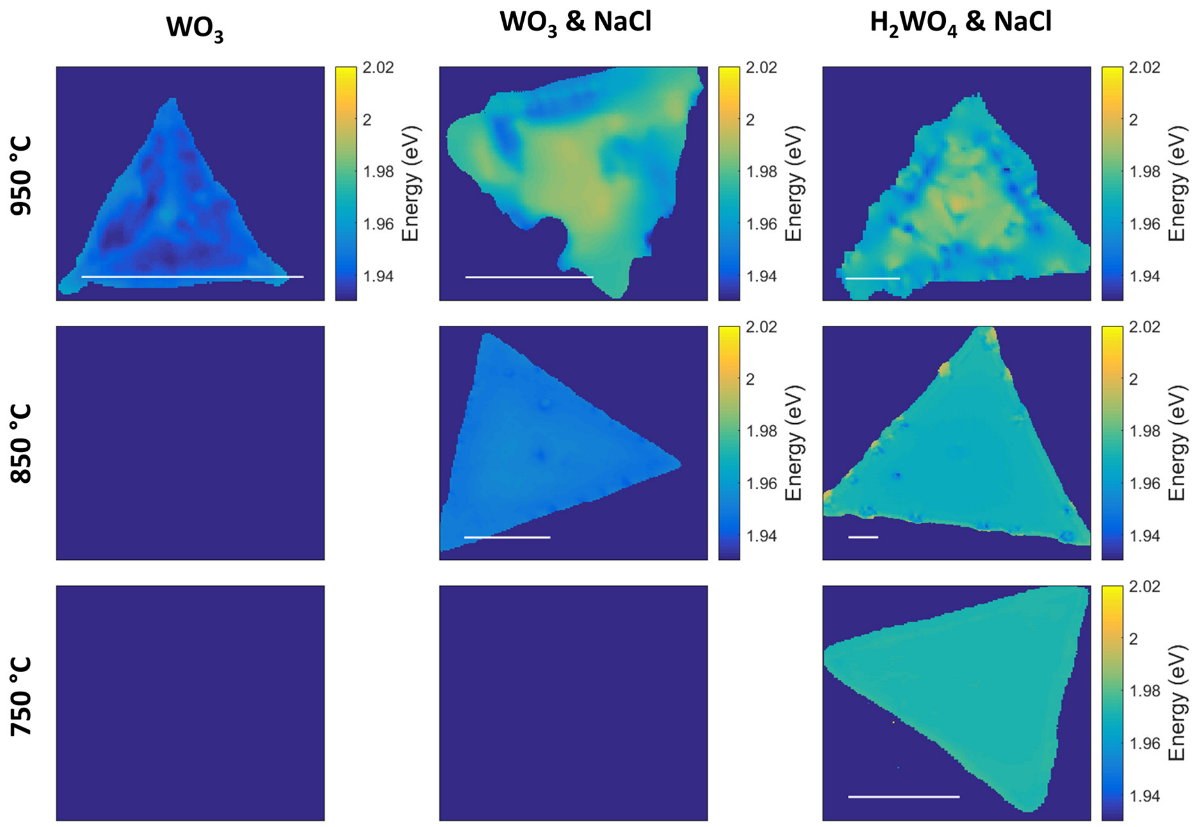

Figure S12. *PL spectroscopy: PL peak position. Scale bar is 10 μm.*

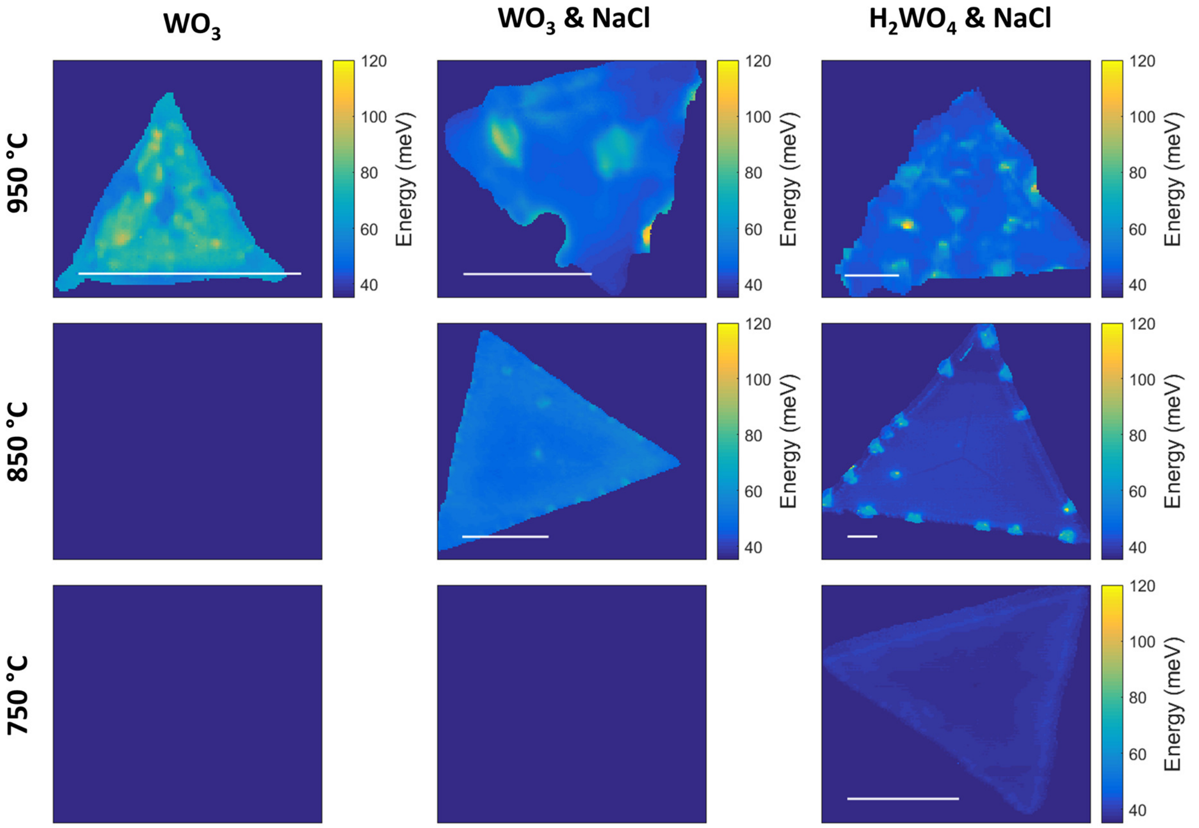

Figure S13. *PL spectroscopy: PL FWHM. Scale bar is 10 µm.*

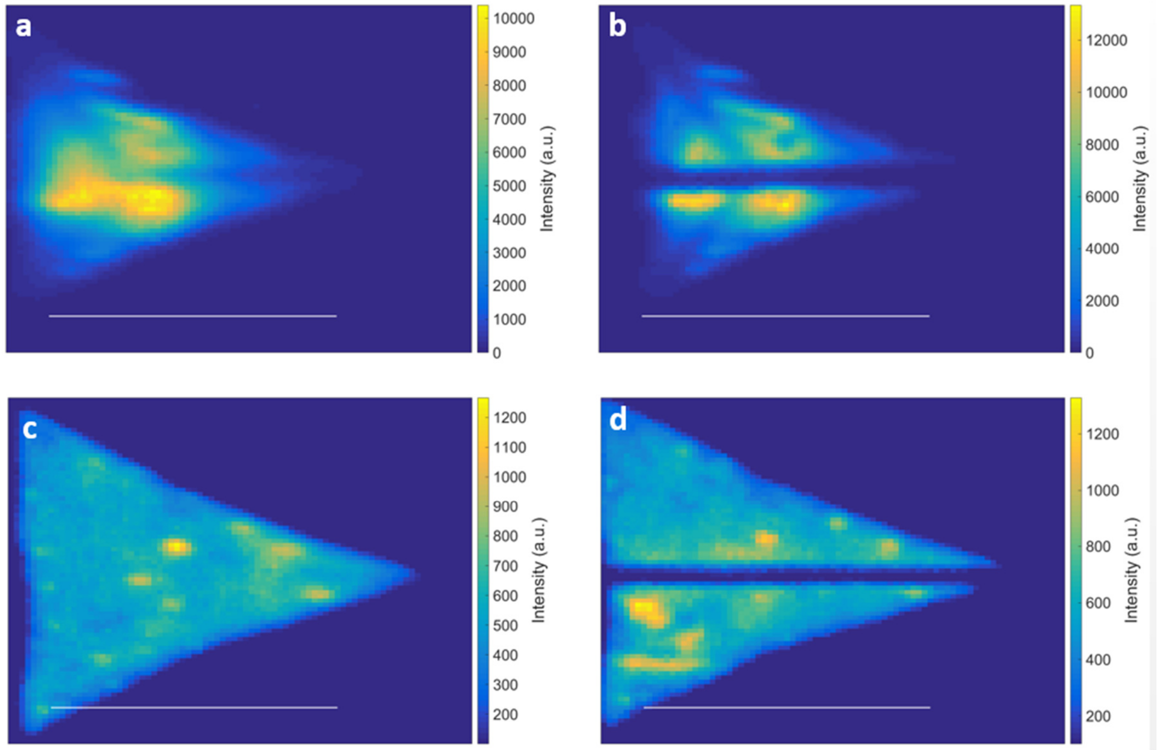

Figure S14. *PL intensity (a) before and (b) after the cutting. 2LA+E^{1}_{2g} Raman intensity (c) before and (d) after the cutting. Scale bar is 10 µm.*

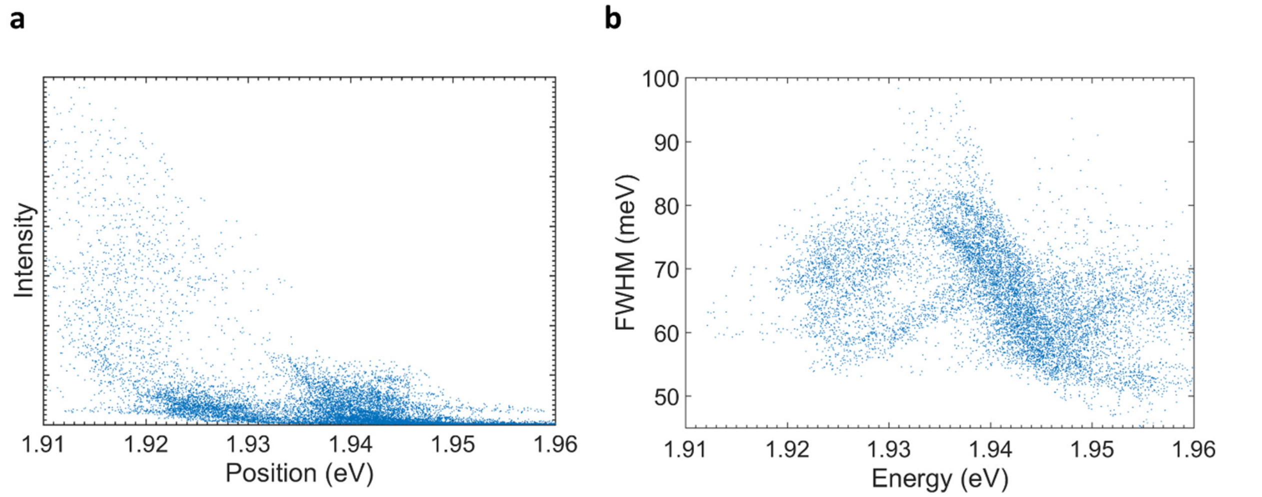

Figure S15. *(a) PL Intensity vs position, (b) PL FWHM vs position of* WS_2 *grown using* WO_3 *at 950 °C*

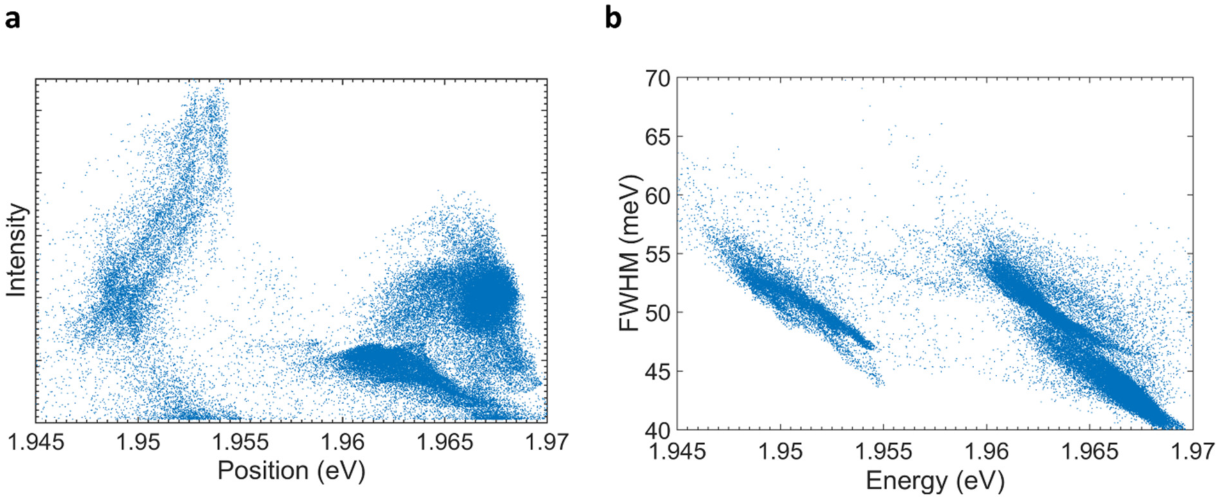

Figure S16. *(a) PL Intensity vs position, (b) PL FWHM vs position of* WS_2 *grown using* WO_3*+NaCl at 850 °C.*

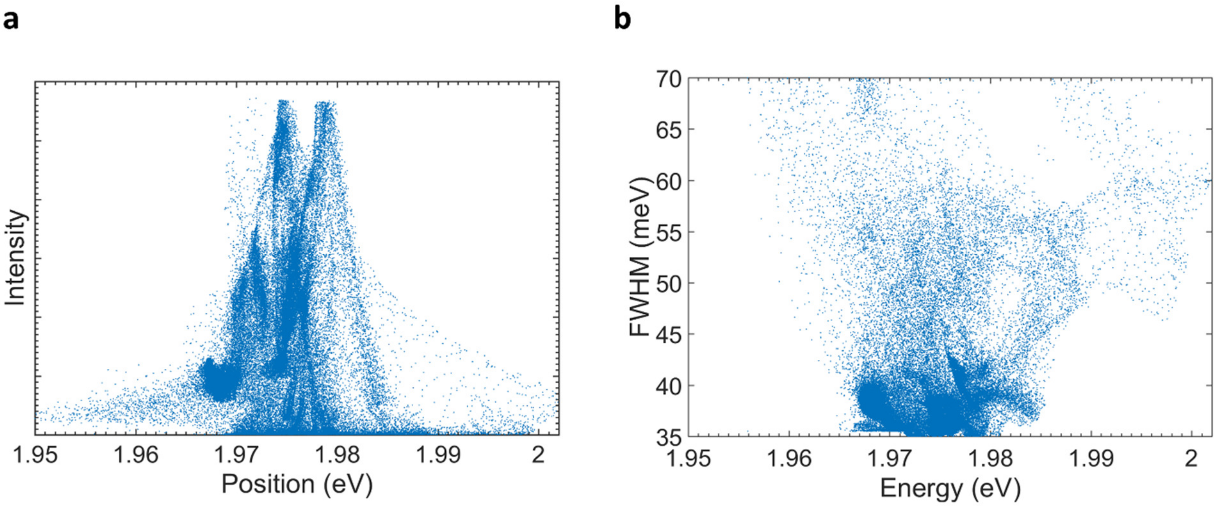

Figure S17. *(a) PL Intensity vs position, (b) PL FWHM vs position of* WS_2 *grown using* H_2WO_4*+NaCl at 850 °C.*

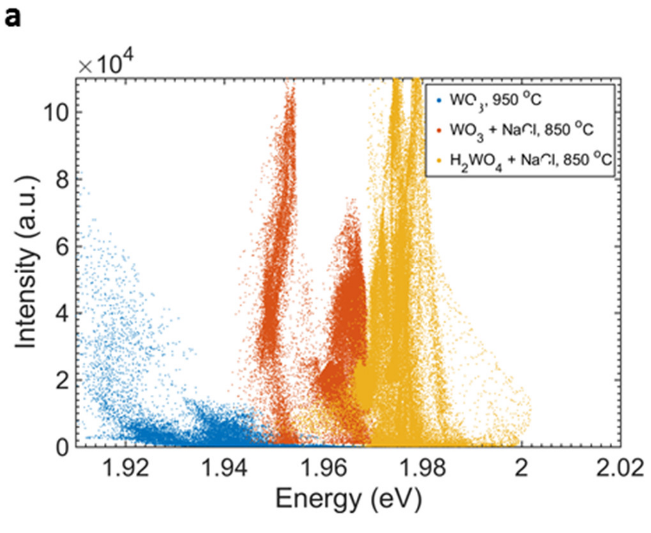

Figure S18. *(a) PL Intensity vs position, (b) PL FWHM vs position of* WS_2 *grown using* WO_3*,* WO_3*+NaCl,* H_2WO_4*+NaCl.*

Electrical Characterization

The gate bias range (Figure 6c) was extended up to the point at which the channel current reached a linear regime, characterised by $I_d = \mu_n C_{ox}(W/L)\,((V_{gs}\text{-}V_{th})V_{ds})$, where μ_n is the electron field-effect mobility, C_{ox} is the oxide dielectric and V_{th} is the threshold voltage. The typical response curves (Figure 6d) at different gate biases for a WS_2 triangle grown using H_2WO_4 and transferred onto a fresh SiO_2 substrate exhibit asymmetry about V_{ds}=0 V which originates from the different electrostatic potential seen at the source and drain electrodes. While the Schottky barrier at the source electrode is pinned by the gate, the drain barrier decreases with negative drain bias, and vice versa. However, as the semiconductor bands become more bent at the semiconductor/metal interface with increasing gate bias, the contribution of tunnelling currents through the barrier becomes more significant and as a result, the contacts become more "Ohmic" in nature.

REFERENCES

(1) Gäggeler, H. W. & Türler, A. *Gas-Phase Chemistry of Superheavy Elements*. *The Chemistry of Superheavy Elements* (2th ed.) (2013).

(2) Wells, A. F. Structural inorganic chemistry (5th ed.). Oxford [Oxfordshire]: Clarendon Press. (1986).

(3) Yang, J., Li, W., Li, J., Sun, D. & Chen, Q. Hydrothermal synthesis and photoelectrochemical properties of vertically aligned tungsten trioxide (hydrate) plate-like arrays fabricated directly on FTO substrates. *J. Mater. Chem.* **22**, 17744−17752 (2012).

(4) Li, S. *et al.* Halide-assisted atmospheric pressure growth of large WSe2 and WS2 monolayer crystals. *Applied Materials Today* **1**, 60−66 (2015).

(5) Ramans, G. M., Gabrusenoks, J. V. & Veispāls, A. A. *et al.* Structure of tungstic acids and amorphous and crystalline WO_3 thin films. *Phys. Status Solidi A* **74**, K41−K44 (1982).

(6) Viswanathan, K., Crystal structure of sodium tetratungstate, $Na_2W_4O_{13}$. *J. Chem. Soc. Dalton Trans.* **20**, 2170−2172 (1974).

(7) Yan, F. *et al.* The ternary system Na_2O–ZnO–WO_3: Compounds and phase relationships. *J. Alloys Compd.* **458** (1-2), 138−143 (2008).

(8) Chang, L. L. Y. & Sachdev, S. Alkali tungstates. Stability relations in the systems alkali monotungstate-tungsten trioxide. *JACS* **58** (7-8), 267−270 (1975).

(9) McKone, J. R., Pieterick, A. P., Gray, H. B. & Lewis, N. S. Hydrogen evolution from Pt/Ru-coated p-type WSe_2 photocathodes. JACS **135**, 223−231 (2013).

(10) Baglio, J., Kamieniecki, E., DeCola, N. & Struck, C. Growth and characterization of n-WS_2 and niobium-doped p-WS_2 single crystals. *J. Solid State Chem.* **49**, 166-179 (1983).

(11) Carmalt, C. J., Parkin, I. P. & Peters, E. S. Atmospheric pressure chemical vapour deposition of WS_2 thin films on glass. *Polyhedron* **22**, 1499−1505 (2003).

(12) Lenz, M. & Gruehn, R. Developments in measuring and calculating chemical vapor transport phenomena demonstrated on Cr, Mo, W, and their compounds. *Chem. Rev.* **97**, 2967−2994 (1997).

(13) Schmidt, P., Binnewies, M., Glaum, R. & Schmidt, M. *Chemical Vapor Transport Reactions – Methods, Materials, Modeling*. *InTech* (2013).

(14) Zhu, B.; Zeng, H.; Dai, J.; Gong, Z. & Cui, X. Anomalously robust valley polarization and valley coherence in bilayer WS_2. *PNAS* **111**, 11606−11611 (2014).

(15) Gutiérrez, H. R. *et al.* Extraordinary room-temperature photoluminescence in triangular WS_2 monolayers. *Nano Lett.* **13**, 3447−3454 (2013).

(16) Rong, Y. *et al.* Controlling sulphur precursor addition for large single crystal domains of WS_2. *Nanoscale* **6**, 12096−12103 (2014).

(17) Hu, P. *et al*. Control of radiative exciton recombination by charge transfer induced surface dipoles in MoS_2 and WS_2 monolayers. *Sci. Rep.* 6:24105 (2016).
(18) Kang, K. N., Godin, K. & Yang, E.-H. The growth scale and kinetics of WS_2 monolayers under varying H_2 concentration. *Sci. Rep.* 5:13205 (2015).
(19) Peimyoo, N. *et al.* Chemically driven tunable light emission of charged and neutral excitons in monolayer WS_2. *ACS Nano* **8**, 11320−11329 (2014).
(20) Tongay, S. *et al.* Defects activated photoluminescence in two-dimensional semiconductors: interplay between bound, charged, and free excitons. *Sci. Rep.* 3:2657 (2013).
(21) Liu, Z. *et al.* Strain and structure heterogeneity in MoS_2 atomic layers grown by chemical vapour deposition. *Nat. Commun.* 5:5246 (2014).
(22) Hui, Y. Y. *et al.* Exceptional tunability of band energy in a compressively strained trilayer MoS_2 sheet. *ACS Nano* **7**, 7126−7131 (2013).
(23) Liu, H. *et al.* Fluorescence concentric triangles: a case of chemical heterogeneity in WS_2 atomic monolayer. *Nano Lett.* ***16***, 5559–5567 (2016).
(24) Zhao, W. *et al*. Evolution of electronic structure in atomically thin sheets of WS_2 and WSe_2. *ACS Nano* **7**, 791−797 (2013).
(25) Shang, J. *et al.* Observation of excitonic fine structure in a 2D transition-metal dichalcogenide semiconductor. *ACS Nano* **9**, 647−655 (2015).
(26) Okada, M. *et al.* Direct chemical vapor deposition growth of WS_2 atomic layers on hexagonal boron nitride. *ACS Nano* **8**, 8273−8277 (2014).
(27) Jo, S., Ubrig, N., Berger, H., Kuzmenko, A. B. & Morpurgo, A. F. Mono- and bilayer WS_2 light-emitting transistors. *Nano Lett.* **14**, 2019−2025 (2014).
